# Emerging Therapeutic Strategies for Neurodegenerative Diseases: A Comprehensive Review of Recent Advances and Future Directions

**DOI:** 10.3390/cells15100928

**Published:** 2026-05-18

**Authors:** Masood Sepehrimanesh, Sarah Victoria Melen, Fatima Yeasmin, Victor Adeleke Ojo, Francisca Walden, Humaira Urmee, Jenna Etheridge, Aruna Kumari Nasu

**Affiliations:** School of Biological Sciences, College of Applied and Natural Sciences, Louisiana Tech University, Ruston, LA 71272, USA

**Keywords:** Alzheimer’s disease, Parkinson’s disease, amyotrophic lateral sclerosis, protein aggregation, neuroinflammation

## Abstract

**Highlights:**

**What are the main findings?**

- Recent advances identify convergent pathogenic mechanisms across neurodegenerative diseases, particularly protein misfolding, neuroinflammation, mitochondrial dysfunction, and impaired proteostasis, as key therapeutic targets.
- Emerging strategies, including gene and cell therapies, RNA-based interventions, immunotherapies, and biomarker-guided precision medicine, show increasing translational potential for disease modification.

**What are the implications of the main findings?**

- Targeting shared molecular pathways across neurodegenerative diseases may enable the development of broad-spectrum or cross-disease therapeutic approaches.
- Integrative, multimodal strategies, combining advanced therapeutics with early diagnosis and personalized interventions, are critical to improving clinical outcomes and overcoming current translational challenges.

**Abstract:**

Neurodegenerative diseases, including Alzheimer’s disease (AD), Parkinson’s disease (PD), and amyotrophic lateral sclerosis (ALS; Lou Gehrig’s disease), represent a growing global health burden characterized by progressive neuronal loss and functional decline. Despite decades of intensive research, effective disease-modifying therapies remain limited, underscoring the urgent need for innovative therapeutic strategies. This review highlights recent advances in the understanding of disease etiology and emerging treatment approaches, with a particular focus on modalities with translational potential. We discussed novel disease-modifying interventions, including gene and cell therapies, RNA-targeting strategies, and immunotherapies aimed at clearing misfolded proteins such as amyloid-β, tau, and α-synuclein. In parallel, we examined the evolving recognition of neuroinflammation and mitochondrial dysfunction as actionable therapeutic targets, alongside progress in precision medicine and biomarker-guided approaches that enable early diagnosis and individualized treatment. Additionally, we summarized developments in repurposed pharmacological agents, neuroprotective compounds, and lifestyle interventions, emphasizing the importance of integrative, multimodal strategies. Across AD, PD, and ALS, convergent molecular mechanisms, including protein misfolding, oxidative stress, and disrupted proteostasis, present opportunities for cross-disease therapeutic targeting. Finally, we addressed key challenges and future directions, including translating preclinical efficacy into clinical success, optimizing CNS-targeted delivery systems, and navigating ethical considerations surrounding gene editing and stem cell therapies.

## 1. Introduction

Alzheimer’s disease (AD), Parkinson’s disease (PD), and amyotrophic lateral sclerosis (ALS) are among the most common neurodegenerative disorders [1], affecting millions worldwide and placing a major burden on healthcare systems due to their progressive and incurable nature [2]. These diseases are characterized by the gradual dysfunction and death of specific neuronal populations, leading to irreversible impairments such as memory loss in AD, motor dysfunction in PD, and muscle paralysis in ALS [2]. Despite decades of research, most currently approved treatments do not stop or reverse disease progression but instead provide only temporary symptomatic relief, highlighting a major unmet clinical need [3]. One reason for this challenge is the highly complex and multifactorial nature of neurodegenerative diseases, which involve interconnected pathological processes rather than a single causative mechanism [2]. Key pathological features include abnormal protein aggregation, such as amyloid-β and tau in AD, α-synuclein in PD, and TDP-43 in ALS, which can spread between neurons and drive disease progression [2]. In addition, mitochondrial dysfunction and oxidative stress impair cellular energy production and increase neuronal vulnerability, contributing to progressive degeneration [4]. Chronic neuroinflammation, driven by activated microglia and astrocytes, further exacerbates neuronal damage and is now recognized as a central contributor to disease progression [5]. Disruptions in autophagy and proteostasis also play a critical role by preventing the proper clearance of misfolded proteins, leading to their toxic accumulation within neurons [2]. Because of this complexity, there has been a shift toward developing therapies that target multiple underlying mechanisms rather than focusing solely on symptoms. Recent advances in pharmacological approaches include small-molecule drugs designed to modulate disease pathways, as well as monoclonal antibodies that target toxic protein aggregates such as amyloid-β [6]. In parallel, stem cell-based therapies are being explored for their potential to replace lost neurons or provide trophic support to damaged neural circuits [7]. Non-pharmacological strategies such as neuromodulation (e.g., deep brain stimulation and transcranial stimulation) are also gaining attention for their ability to modulate neural activity and improve functional outcomes [7]. Furthermore, cutting-edge approaches such as gene therapy aim to correct or silence disease-causing genes, offering the potential for long-term or even curative effects [6]. Emerging fields like bioelectromagnetics and neuroprotective compound development are also being investigated for their ability to protect neurons and slow disease progression [7].

This comprehensive review evaluates recently reported treatments for AD, PD, and ALS, focusing on their mechanisms of action, clinical efficacy, and translational potential. By synthesizing current advancements, we aim to provide insights into the evolving therapeutic landscape, highlighting both challenges and future directions for research and clinical applications.

## 2. Methods

Although this study is presented as a comprehensive review, a structured literature search strategy was employed to enhance transparency and rigor. Relevant articles were identified using multiple databases, including PubMed, Google Scholar, ScienceDirect, Elsevier, and Springer. The search strategy included combinations of the following keywords: “Parkinson’s disease (PD),” “Alzheimer’s disease (AD),” “amyotrophic lateral sclerosis (ALS),” along with treatment-related terms such as “treat*” “therapy*” and “efficacy” in the title and or Abstract.

The timeframe for the literature search was restricted to studies published between 2020 and 2026. Inclusion criteria consisted of original research articles focusing on therapeutic approaches, including newly developed treatments, modifications of existing therapies, or evaluations of current treatment strategies for PD, AD, and ALS, with particular emphasis on treatment efficacy. Exclusion criteria included review articles, case reports, studies not related to treatment, articles published outside the specified timeframe, non-English publications, and studies lacking sufficient scientific rigor or relevance to the objectives of this review.

## 3. Alzheimer’s Disease

### 3.1. Epidemiology and Pathomechanisms

AD is a progressive neurodegenerative disorder characterized by cognitive decline, behavioral changes, and functional impairment [8,9]. It is the leading cause of dementia, accounting for approximately 60–80% of all dementia cases worldwide [10]. In 2019, an estimated 5.8 million Americans were affected by AD [11], and this number is projected to rise substantially due to increased life expectancy and aging populations. By 2020, approximately 50 million people globally were living with dementia, with projections suggesting an increase to 152 million by 2050 [12]. The disease is more prevalent in women than men, likely due to differences in lifespan and hormonal influences [10]. AD is a multifactorial disease influenced by genetic, environmental, and lifestyle factors [12]. The two primary types of AD are sporadic (late-onset) AD and familial (early-onset) AD. Sporadic AD, the most common form, primarily affects individuals aged 65 and older and is influenced by aging, cardiovascular diseases, diabetes, obesity, and environmental exposures [8]. In contrast, familial AD is a rare genetic variant accounting for less than 5% of cases and is associated with mutations in the gene coding amyloid precursor protein (APP), Presenilin (PSEN)-1 and PSEN-2 genes, leading to abnormal amyloid-beta (Aβ) production and accumulation [13]. The age of symptom onset varies, with initial manifestations typically appearing in the fifties [14,15,16] or sixties [17,18], while the average age of AD diagnosis is in the early seventies [17,18]. Pathophysiologically, AD is primarily characterized by Aβ plaques and neurofibrillary tangles composed of hyperphosphorylated tau proteins [13]. The amyloid hypothesis posits that Aβ peptides, derived from APP, form extracellular plaques that disrupt synaptic function, trigger neuroinflammation, and lead to neuronal death [19]. Conversely, the tau hypothesis suggests that intracellular tau tangles impair axonal transport and contribute to neurodegeneration [13]. Other proposed mechanisms include cholinergic dysfunction, mitochondrial impairment, and neuroinflammation [10]. Increased Aβ plaques and tau tangles have been detected in pre-symptomatic AD patients through neuroimaging techniques such as positron emission tomography (PET) and magnetic resonance imaging (MRI) [17,18], which are crucial for understanding disease progression and biomarker fluctuations.

Genetic risk factors play a crucial role in AD development, with the apolipoprotein E (APOE ε4) allele significantly increasing susceptibility to late-onset AD [13]. Additionally, lifestyle factors such as alcohol consumption, depression, smoking, physical inactivity, and poor diet have been strongly associated with AD progression [20]. Psychological disturbances, assessed through the Neuropsychiatric Inventory (NPI) [9,15] and the Geriatric Depression Scale (GDS) [9], have been studied in limited cases, yet they remain critical in understanding and managing AD symptoms. Currently, there is no cure for AD, and available treatments primarily focus on symptom management. The most commonly prescribed pharmacological agents are cholinesterase inhibitors, such as donepezil, rivastigmine, and galantamine, as well as N-methyl-D-aspartate receptor (NMDAR) antagonists like memantine [8,21,22]. Emerging therapies, including anti-amyloid and anti-tau treatments, alongside nanotechnology-based drug delivery systems, provide new avenues for potentially slowing disease progression. Non-pharmacological interventions, such as repetitive transcranial magnetic stimulation (rTMS) [15,23], aerobic exercise [11], and music therapy [9], have demonstrated cognitive benefits and are promising adjuncts to conventional treatments. Extensive research efforts continue to explore novel therapeutic targets, with recent studies shifting focus from reducing amyloid and tau accumulation to addressing neuroinflammation and other pathological processes [14,16,17,18]. The findings of Dubois et al. and Liu et al. suggest that modulating inflammatory pathways may provide a more effective strategy for combating AD progression. Future research should emphasize biomarker-based studies, utilizing advanced imaging techniques and molecular analyses to refine early diagnosis and therapeutic approaches. The integration of cognitive and psychological interventions, such as social engagement and structured activities like cycling [11] and music therapy [9], may offer synergistic benefits when combined with pharmacological treatments.

### 3.2. New Therapeutic Reports

**Figure 1:**
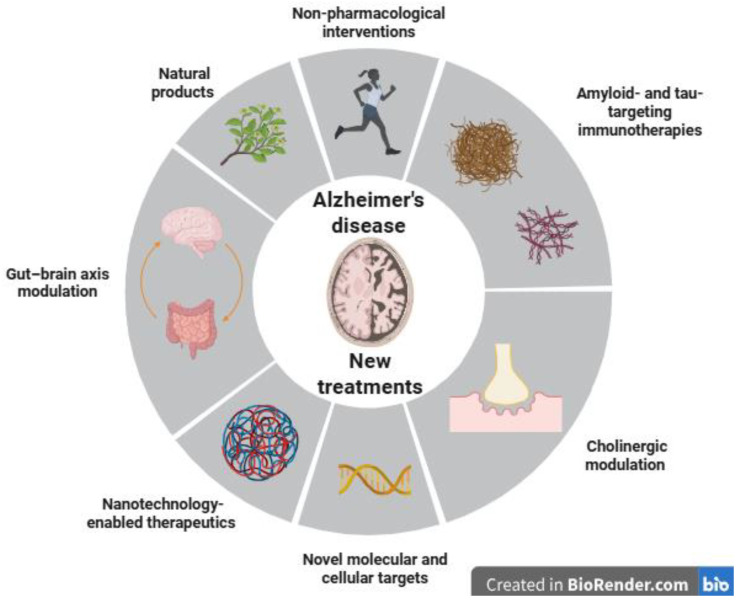
Emerging therapeutic strategies for Alzheimer’s disease (AD).

In recent years, advances in our understanding of AD pathophysiology have driven the development of diverse and innovative therapeutic strategies. These emerging approaches go beyond traditional symptomatic treatments, aiming to modify disease progression through molecular, cellular, and systemic mechanisms. The following sections highlight several promising avenues, including pharmacological, immunological, technological, and lifestyle-based interventions designed to address the multifaceted nature of AD (Figure 1). In Table 1, the novel treatments are organized under an alternative categorization, with concise descriptions of each study’s main features.

#### 3.2.1. Cholinergic Modulation and Symptomatic Therapies

Cholinergic modulation remains a cornerstone in the symptomatic management of neurodegenerative diseases, particularly AD, where acetylcholine deficiency has a significant role to play in cognitive decline. Developing advancements revolve around increasing cholinergic transmission through novel acetylcholinesterase (AChE) inhibitors (AChE-I), designing improved pharmacokinetic profiles that maximize blood–brain barrier (BBB) penetration, reduce unwanted effects, and sustain therapeutic activity for an extended period. Hussain et al. investigated benzoxazole-based oxazole derivatives as potential cholinesterase inhibitors for AD [8]. Current treatments, such as donepezil and rivastigmine, offer only short-term symptomatic relief and have adverse effects. This study aimed to develop more effective inhibitors targeting AChE and butyrylcholinesterase (BuChE). Nineteen novel benzoxazole-1,3-oxazole derivatives were synthesized and characterized using high-resolution electron ionization mass spectrometry (HREI-MS) and nuclear magnetic resonance (NMR) spectroscopy. Enzyme inhibition assays revealed half maximal inhibitory concentration (IC_50_) values as low as 0.90 µM for AChE and 1.10 µM for BuChE, with analogs 11 and 18 outperforming donepezil. Molecular docking confirmed favorable enzyme-ligand interactions, supporting their potential efficacy. These findings highlight benzoxazole-based oxazole derivatives as promising candidates for AD therapy, warranting further optimization for improved efficacy and safety. The pharmacokinetics of donepezil, a widely used AChE-I for AD, have been investigated by analyzing its cerebrospinal fluid (CSF) and plasma concentrations over a 24 h period [24]. Given ongoing debate regarding optimal dosing, understanding donepezil’s central nervous system (CNS) penetration is crucial for improving treatment strategies. The study included 40 AD patients who had been on a 10 mg/day regimen for at least three months. Participants were assigned to one of four sampling groups (6, 12, 18, and 24 h post-dose) for CSF and plasma collection. Donepezil concentrations were measured using high-performance liquid chromatography (HPLC), and statistical analyses (Kruskal–Wallis and Mann–Whitney U tests) assessed variations across time points. Results showed that while CSF and plasma concentrations remained relatively stable, the CSF/plasma ratio significantly increased at 24 h compared to 6 h (*p* < 0.005) and 12 h (*p* < 0.05). These findings suggest that donepezil continues accumulating in the CNS even after plasma levels stabilize around 20 h, indicating prolonged absorption into the brain. This study highlights the CSF/plasma ratio as a potential biomarker for optimizing donepezil dosing, allowing for individualized dose adjustments to maximize cognitive benefits while minimizing side effects. Further research is needed to evaluate higher doses and alternative formulations for improved AD treatment strategies. Another study investigated whether increasing the dosage of FDA-approved donepezil could extend its efficacy in AD treatment [22]. Given the drug’s limited long-term effects, a 24-week, double-blind study was conducted across 219 sites worldwide, involving 1371 patients with moderate-to-severe AD, as determined by MMSE scores. Participants were randomly assigned to receive either the standard 10 mg/day dose or an increased 23 mg/day dose. Cognitive function was assessed using the Severe Impairment Battery (SIB), while global function was evaluated with Clinician’s Interview-Based Impression of Change plus Caregiver Input (CIBIC-plus). Results indicated a significant cognitive benefit in the 23 mg/day group compared to the 10 mg/day group, particularly among patients with more severe AD. However, CIBIC-plus scores did not show significant differences. Adverse effects, including diarrhea, nausea, and vomiting, were more frequent in the higher-dose group. These findings suggest that an increased donepezil dosage may provide greater cognitive benefits, particularly in severe AD cases. Future studies should further explore dosage optimization, although biomarker analysis was deemed less critical for this investigation, given the extensive existing research on donepezil’s mechanisms.

#### 3.2.2. Amyloid- and Tau-Targeting Immunotherapies

Amyloid- and tau-immunotherapies are promising disease-modifying therapies that act on AD pathological mechanisms with the aim of halting or even reversing them. Both active and passive immunization strategies are being optimized to enhance specificity, reduce neuroinflammatory side effects, and provide effective amyloid-β aggregates and brain clearance of hyperphosphorylated tau proteins. A major challenge highlighted by recent AD immunotherapy trials is the discrepancy between successful target engagement and limited clinical efficacy, particularly in therapies directed against amyloid-β and tau pathology. Several monoclonal antibodies demonstrated measurable reductions in pathological biomarkers, confirming that these agents effectively interact with their intended molecular targets. However, these biomarker improvements were often accompanied by only modest or statistically insignificant benefits in cognitive and functional outcomes, suggesting that pathological protein clearance alone may not be sufficient to halt or reverse disease progression. One possible explanation is that AD pathology develops over decades before clinical symptoms become apparent, meaning that therapeutic intervention during symptomatic stages may occur after irreversible neuronal loss, synaptic dysfunction, and widespread network degeneration have already taken place. Furthermore, AD is increasingly recognized as a multifactorial disorder involving interconnected mechanisms, all of which may continue to drive neurodegeneration independently of amyloid or tau burden. The inconsistency between biomarker responses and clinical outcomes also raises important questions regarding the validity of amyloid reduction as a surrogate endpoint for therapeutic success. These findings support the emerging concept that therapeutic efficacy in AD may depend heavily on treatment timing, duration, and patient stratification based on biomarker profiles, emphasizing the need for precision medicine approaches and combination therapies targeting multiple pathological pathways simultaneously to achieve more substantial and durable clinical benefits.

Sperling et al. conducted a phase 3 trial investigating solanezumab, a monoclonal antibody targeting Aβ accumulation, as a potential treatment for early AD [17]. Since Aβ deposition begins before clinical symptoms appear, the study aimed to determine whether solanezumab could slow cognitive decline by reducing amyloid buildup. This double-blind, placebo-controlled trial followed 1169 early AD patients over 4.5 years, assessing cognitive function using the Clinical Dementia Rating (CDR), Mini-Mental State Examination (MMSE), and Wechsler Memory Scale Logical Memory Delayed Recall (LMDR). Amyloid PET imaging and MRI were used to monitor hippocampal and gray matter volume, while the Preclinical Alzheimer Cognitive Composite (PACC) measured cognitive outcomes every four weeks. Despite extensive monitoring, results showed no significant difference in PACC scores between the solanezumab and placebo groups. Amyloid levels continued to increase rather than decline, and there was no measurable reduction in Aβ plaques. The study also faced disruptions due to the COVID-19 pandemic, which may have impacted assessments and treatment schedules. The findings suggest that solanezumab did not effectively slow AD progression. The researchers speculate that a dosage adjustment in 2017, aimed at accommodating a larger participant pool, may have altered Aβ clearance dynamics, potentially contributing to the lack of observed therapeutic benefit. Further research is needed to refine amyloid-targeting strategies for AD.

A study investigating the effects of lecanemab, an antibody targeting soluble Aβ fibrils, in early AD [14]. Soluble Aβ fibrils are considered more neurotoxic than insoluble plaques, and this phase 3, double-blind trial aimed to assess lecanemab’s potential in reducing amyloid accumulation and cognitive decline. The study enrolled 1795 patients over 18 months, with PET imaging used to monitor amyloid levels in CSF. Cognitive and functional assessments included the Clinical Dementia Rating-Sum of Boxes (CDR-SB), MMSE, Alzheimer’s Disease Assessment Scale-Cognitive Subscale (ADAS-cog14), AD Composite Score (ADCOMS), and the AD Cooperative Study-Activities of Daily Living Scale for Mild Cognitive Impairment (ADCS-MCI-ADL). Results showed no significant improvement in CDR-SB and ADCOMSs for lecanemab-treated patients compared to placebo, although ADAS-cog14 and ADCS-MCI-ADL scores favored lecanemab. While PET scans confirmed selective reduction of soluble Aβ fibrils, the clinical significance of this reduction remains uncertain. Adverse events were more frequent in the lecanemab group during the first four months but stabilized over time. Serious effects included infusion-related reactions and nearly double the incidence of hemorrhages compared to placebo (17.3% vs. 9.0%). Tau PET and MRI data were not fully analyzed at the time of publication. These findings suggest that while lecanemab effectively reduces soluble Aβ fibrils, its clinical benefits remain unclear, warranting further investigation into its long-term efficacy and safety in AD treatment. Long-term follow-up analyses of lecanemab showed sustained reductions in amyloid burden alongside continued slowing of cognitive decline in early AD [25]. The 2025 data highlighted that prolonged treatment maintained clinical benefits beyond the initial trial period, which suggested a chronic disease-modifying mechanism rather than transient symptomatic relief. Furthermore, the study provided updated safety characterization, indicating that rates of amyloid-related imaging abnormalities (ARIA) were manageable with appropriate monitoring. Collectively, these findings strengthened the clinical validity of amyloid-targeting strategies and underscored the importance of early and continuous therapeutic intervention to achieve meaningful disease modification. Florian et al. investigated the efficacy of tilavonemab, an anti-tau monoclonal antibody, in slowing AD progression [18]. Unlike Aβ, which plateaus in late-stage AD, tau pathology continues to worsen, making it a critical target for intervention. This double-blind, 96-week study assessed whether tilavonemab could reduce insoluble tau and impact disease progression. A total of 453 early AD patients, confirmed via clinical scores (CDR, MMSE, RBANS-DMI) and amyloid PET scans, were randomized to receive tilavonemab (300 mg, 1000 mg, or 2000 mg) or placebo via infusion every four weeks. Biomarker assessments included MRI for tau burden, PET for amyloid levels, and CSF analysis for neurofilament light chain (NfL). Cognitive and functional decline were measured using CDR-SB, ADAS-cog, ADCOMS, ADCS-MCI-ADL-24, and MMSE. Results showed no significant differences between tilavonemab and placebo across all clinical and imaging outcomes. Amyloid burden remained unchanged, and volumetric MRI failed to detect treatment-related effects. While free tau levels significantly decreased in week 12 in the 1000 mg and 2000 mg groups, and total tau decreased only in the 300 mg group, these findings did not translate into cognitive benefits. They concluded that while tilavonemab demonstrated target engagement, its lack of clinical efficacy led to the discontinuation of further trials.

Extended analyses of the TRAILBLAZER-ALZ 2 trial demonstrated that donanemab, a monoclonal antibody targeting deposited amyloid-β plaques, exerted sustained disease-modifying effects [26]. Follow-up data from 2025 confirmed that early amyloid clearance correlated with slower cognitive and functional decline, particularly among patients with low-to-intermediate tau burden at baseline. These results reinforced the clinical utility of biomarker-guided patient stratification, as therapeutic efficacy proved strongly dependent on disease stage and tau pathology. While ARIA remained a notable adverse effect, the long-term data supported donanemab as a promising therapy that shifted treatment paradigms toward early intervention and precision medicine in AD.

#### 3.2.3. Novel Molecular and Cellular Targets

Recent research has pointed out an array of novel molecular and cellular targets that unveil treatment possibilities beyond traditional pathways in neurodegenerative disorders. These novel targets offer new avenues for intervention toward preventing or halting neuronal loss. Musardo et al. investigated the role of a disintegrin and metalloproteinase 10 (ADAM10) in AD pathology, focusing on its endocytosis as a potential therapeutic target [19]. ADAM10 cleaves the APP into sAPPα, a neuroprotective fragment that prevents Aβ plaque formation. This study explored whether inhibiting ADAM10 endocytosis using a cell-penetrating peptide (PEP3) could enhance synaptic function and slow disease progression. The authors developed four peptide inhibitors (PEP1-PEP4) to disrupt the ADAM10/Adaptor protein complex 2 (AP2) interaction that regulates endocytosis. Among them, PEP3 significantly increased synaptic ADAM10 retention and sAPPα production without altering total ADAM10 levels. Electrophysiological recordings demonstrated enhanced synaptic plasticity, and biochemical assays confirmed its role in neuroprotective APP processing. In vivo, PEP3 improved cognitive performance and synaptic spine density in 6-month-old APP/presenilin-1(PSEN-1) transgenic mice but failed to reverse cognitive decline in 12-month-old mice, suggesting its efficacy depends on early intervention. This study identifies PEP3 as a promising early-stage AD therapeutic that enhances synaptic ADAM10 and neuroprotective APP processing. Future research should focus on clinical translation, long-term safety, and optimized drug delivery strategies to advance PEP3 as a potential treatment for AD.

As a critical immune receptor within the CNS, triggering receptor expressed on myeloid cells-2 (TREM2) was shown to regulate microglial survival, phagocytic activity, and the orchestration of neuroinflammatory responses. While the therapeutic targeting of TREM2 was historically limited to antibody-based approaches with poor CNS penetration and manufacturing constraints, Nada et al. identified S9 as a first-in-class submicromolar small-molecule agonist. S9 demonstrated robust TREM2 agonism by inducing proximal Syk phosphorylation, activating downstream nuclear factor of activated T cells (NFAT) transcriptional signaling, and enhancing both APOE internalization and microglial phagocytic capacity. Pharmacokinetic profiling revealed that S9 possessed significantly improved drug-likeness over its precursor, characterized by a 7-fold increase in aqueous solubility, superior metabolic stability, and a 9-fold enhancement in the hERG safety margin. Functional validation in human iPSC-derived microglia and neuron-microglia co-culture models further confirmed that S9 suppressed Aβ-induced IL-1β secretion and preserved synaptic integrity, as evidenced by sustained postsynaptic density protein 95 (PSD95) expression. Collectively, their findings established S9 as a potent, orally bioavailable TREM2 modulator with the potential to address the unmet therapeutic gap in AD precision medicine [27].

An alternative approach to AD treatment was explored, shifting focus away from amyloid-targeting therapies due to their lack of demonstrated clinical efficacy [16]. Instead, they investigated the role of mast cells and glial activity in AD progression, testing masitinib, a tyrosine kinase inhibitor that modulates these cells. This phase 3, double-blind, randomized trial included 718 mild-to-moderate AD patients over 24 weeks. Participants received either a placebo, masitinib at 4.5 mg/kg/day, or a titrated dose of 6.0 mg/kg/day. Cognitive function and dementia progression were assessed using ADAS-cog, ADCS-ADL, MMSE, CDR, and CIBIC-plus. Results showed a significant reduction in cognitive decline in the masitinib group, as measured by ADAS-cog, compared to the placebo. However, ADCS-ADL and titrated-dose results were not statistically significant. The optimal dosage was determined to be 4.5 mg/kg/day, as the higher dose led to increased adverse effects. Notably, no biomarker analysis was conducted to confirm whether masitinib directly targeted mast cells and glial activity. Dubois et al. concluded that this study represents the first successful phase 3 trial of a tyrosine kinase inhibitor for AD. However, the underlying mechanisms remain unverified, necessitating further investigation to confirm masitinib’s specific biological effects in AD treatment.

Although gene therapy targeting APOE has gained attention as a potential treatment for AD, Yang and colleagues introduced an innovative inducible adeno-associated virus (AAV) system showing that timing plays a decisive role in therapeutic success. Using 5×famialial AD mice, they found that continuous expression of APOE4 intensified cognitive impairment, neuroinflammation, and amyloid-β (Aβ) accumulation. In contrast, prolonged expression of APOE2 alongside suppression of APOE4 effectively mitigated these pathological features and improved cognitive performance. Notably, short-term APOE2 replacement not only failed to confer benefits but unexpectedly aggravated both behavioral deficits and disease pathology, which the authors attributed to a “rebound-adaptation” effect. Further transcriptomic analysis revealed Ras-related protein in brain 24 (RAB24), a key regulator of autophagic trafficking, as an important contributor to this response. RAB24 levels were elevated under APOE4 expression and during transient APOE2 exposure, whereas its downregulation during sustained APOE2 treatment was necessary to reestablish Aβ clearance and maintain cholesterol balance. Altogether, these results highlight that the effectiveness of APOE-directed therapies depends on duration-specific molecular changes and identify RAB24 as a potential therapeutic target for improving lysosomal function and metabolic regulation in AD [28].

#### 3.2.4. Nanotechnology-Enabled Therapeutics and Drug Delivery Systems

Nanotechnology-based drugs and delivery systems are transforming the therapeutic landscape of neurodegenerative diseases by optimizing targeted delivery across the BBB. Such advanced platforms optimize drug stability, bioavailability, and controlled release to allow targeted regulation of disease mechanisms with low systemic side effects. El-Hawwary et al. explored the potential of green-synthesized zinc oxide nanoparticles (ZnO NPs) derived from *Sabal blackburniana* as an alternative treatment for AD [29]. While donepezil, a common AChE-I, is effective, its significant side effects limit its therapeutic use. This study aimed to determine whether eco-friendly ZnO NPs offer a safer alternative. ZnO NPs were synthesized using methanolic extracts from the leaves, fruits, and pollen of *S. blackburniana* and characterized through UV-visible spectroscopy, transmission electron microscopy (TEM), X-ray diffraction (XRD), Fourier transform infrared spectroscopy (FTIR), and zeta potential measurements. The anti-Alzheimer potential was assessed via AChE inhibition using the Ellman method, comparing ZnO NPs and plant extracts with donepezil. Liquid chromatography-mass spectrometry (LC-MS) identified bioactive compounds contributing to inhibition. The results demonstrated strong AChE inhibitory activity, with ZnO NPs from leaves exhibiting an IC_50_ of 63.78 ng/mL, comparable to donepezil (IC_50_ = 50.7 ng/mL). Molecular docking revealed that non-glycosylated flavonoids, such as luteolin and catechin, formed stable interactions with AChE’s active site, contributing to inhibition. These findings suggest that ZnO NPs could serve as a sustainable and effective AD treatment with fewer side effects than conventional drugs. Further research is needed to validate their clinical potential and optimize their therapeutic application.

A transdermal drug delivery system was explored before for donepezil and now for huperzine A (Hup A), a potent AChE-I with potential for AD treatment 30. Recent studies have further strengthened the relevance of microneedle-based delivery for Alzheimer’s therapeutics by demonstrating successful transdermal administration of donepezil using hydrogel-forming, dissolving, and hybrid microneedle platforms. These systems have shown improved skin permeability, sustained drug release, and enhanced systemic exposure in preclinical models, highlighting their translational potential for overcoming limitations of oral donepezil therapy. A subcutaneous implantable microneedle system has been reported to provide sustained delivery of donepezil with improved pharmacodynamic outcomes in vivo, while dissolving microneedle patches have demonstrated favorable pharmacokinetics compared to oral dosing. More recently, hybrid microneedle–microparticle systems have been developed to further extend intradermal release profiles, supporting prolonged therapeutic exposure and reduced dosing frequency [33,34,35]. Given Hup A’s low bioavailability, short half-life, and frequent dosing requirements, a dissolving microneedle patch (DMNP) was developed as a more efficient and less invasive alternative to oral administration. The study assessed DMNPs through scanning electron microscopy, mechanical compression testing, and in vivo skin penetration analysis. Results showed that DMNPs effectively penetrated the skin and dissolved within 5 min, enabling sustained drug release. Pharmacokinetic studies demonstrated a fivefold increase in Hup A half-life and a twofold increase in drug exposure compared to oral dosing, while reducing peak plasma concentrations, thereby minimizing dosage spikes and potential side effects. Over 80% of Hup A was released within three days. Pharmacodynamic evaluations confirmed that DMNP-delivered Hup A significantly improved cognitive function in AD models, exhibited strong antioxidant properties, and enhanced cholinergic activity. These findings suggest that DMNPs offer a promising, patient-friendly alternative for sustained Hup A delivery, potentially improving both efficacy and treatment adherence in AD [30].

Liu et al. explored an alternative to Aβ-targeted therapies by developing a nanozyme-based approach to mitigate neuroinflammation in AD [31]. Their strategy involved using carbon monoxide (CO) as a neuroprotective agent, delivered via a metal–organic framework (MOF) designed for CO loading and controlled release. The MOF was synthesized using microwave irradiation and characterized by TEM and scanning electron microscopy (SEM), revealing high porosity and surface area suitable for drug loading. A CO-releasing compound (Fla) was incorporated into the MOF, with FTIR and XRD confirming structural stability. To enhance targeting, a neutrophil coating was added. In vivo testing involved Aβ-induced AD model mice, with memory recall assessed using the Morris water maze (MWM). Fluorescence imaging demonstrated precise targeting of inflamed brain cells, with CO release reducing inflammatory signals. Mice treated with the neutrophil-coated metal–organic framework (NeuMOF)/Fla formulation exhibited improved cognitive performance and showed biocompatibility with low toxicity. These findings suggest that CO-loaded nanozymes offer a promising approach for addressing neuroinflammation in AD, potentially complementing or improving upon existing Aβ-targeted therapies.

A hydrogel-based drug delivery system was developed to enhance the controlled release of galantamine hydrobromide (GAH) and reduce its gastric side effects in AD treatment [21]. Using simulated gastric and intestinal fluids (SGF and SIF), the study evaluated the slow-release behavior of GAH-loaded microcapsules. Mesoporous silica nanoparticles (MSNPs) were synthesized, loaded with GAH, and encapsulated via an electrospray method to form hydrogel microcapsules. SEM analysis confirmed their spherical morphology and porosity, while biocompatibility assays demonstrated their safety. In vitro studies showed that hydrogel microparticles facilitated a sustained release of GAH over 4–6 h. In vivo testing using the MWM revealed significant memory improvement in mice treated with GAH-loaded hydrogels compared to controls. ELISA analysis further showed reduced AChE activity, indicating neuroprotection. These findings suggest that hydrogel-based drug delivery offers a promising approach for sustained and targeted GAH administration in AD management.

#### 3.2.5. Natural Products and Phytochemical-Based Neuroprotection

Plant-derived natural products and phytochemical treatments are also being contemplated for their neuroprotective potential in neurodegenerative diseases. Plant-derived bioactive molecules, such as polyphenols, alkaloids, and terpenoids, have antioxidant, anti-inflammatory, and anti-amyloidogenic activities and offer a complementary approach to conventional pharmacologic therapy. Wang et al. investigated the pharmacological potential of secondary metabolites from *Dictyostelium discoideum* as multi-target therapeutics for AD [32]. These natural compounds were analyzed for their ability to modulate key AD-related pathways, particularly neuroinflammation and oxidative stress, which contribute to disease progression. Bioactive metabolites were identified by cross-referencing AD-associated genes from GeneCards, DisGeNET, and the Chemical and Toxicology Database with *D. discoideum* metabolites using Swiss Target Prediction. Gene ontology (GO) and Kyoto Encyclopedia of Genes and Genomes (KEGG) pathway enrichment analysis determined the biological significance of protein–protein interactions. Molecular docking studies evaluated binding affinities between these compounds and neuroinflammatory targets, including cyclooxygenase (COX)-2, mitogen-activated protein kinase (MAPK)-8, heat shock protein 90 alpha family class B member 1 (HSP90AB1), and mechanistic target of rapamycin (MTOR). Results demonstrated strong interactions between these metabolites and inflammation-associated cytokines (interleukin (IL)-1, IL-2, tumor necrosis factor (TNF)-α), suggesting their potential to suppress neuroinflammation and Aβ plaque formation. Notably, PQA11 exhibited high binding affinity to COX-2, highlighting its potential to reduce oxidative stress and inflammation, as confirmed by molecular docking and dynamics simulations. These findings support *D. discoideum* metabolite derivatives as promising multi-target candidates for AD therapy. Further in vitro and in vivo studies are needed to validate their efficacy and potential as plant-based therapeutic agents for reducing neuroinflammation and cognitive decline in AD.

#### 3.2.6. Gut–Brain Axis Modulation

Gut–brain axis modulation has emerged as a very promising strategy for the modulation of neurodegenerative disease progression. By targeting bidirectional communication between the gut microbiota and the CNS, interventions such as probiotics, prebiotics, and diet seek to inhibit neuroinflammation, improve metabolic homeostasis, and support neuronal health. Den et al. conducted a meta-analysis of randomized controlled trials (RCTs) to assess the effects of probiotic supplementation on cognitive function, inflammation, and oxidative stress in individuals with AD or mild cognitive impairment (MCI) [12]. Given the link between gut microbiota and neurodegeneration, probiotics have emerged as potential modulators of inflammation and oxidative stress. A systematic review of EMBASE, PubMed, and the Cochrane Library identified five eligible RCTs with 297 participants. Standardized mean differences (SMDs) were calculated to compare cognitive outcomes and biomarker changes between probiotic and placebo groups, with statistical analyses for heterogeneity and publication bias. Results showed that probiotics significantly improved cognitive function (SMD = 0.37) and reduced malondialdehyde and high-sensitivity C-reactive protein levels, indicating lower oxidative stress and inflammation. While probiotics represent a promising, low-cost intervention for cognitive decline, effects vary by patient subgroup and probiotic strain. Larger, long-term studies are needed to confirm findings and elucidate underlying mechanisms, which suggest probiotics as a potential gut-targeted therapy for neurodegeneration.

#### 3.2.7. Non-Pharmacological Interventions and Neuromodulation

Non-pharmacological interventions and neuromodulation offer new strategies to traditional drug interventions for neurodegenerative disease. Therapeutic techniques such as cognitive training, physical exercise, TMS, and deep brain stimulation are tailored to enhance neural plasticity, repair diseased circuits, and augment cognitive and motor function. Moussavi et al. investigated the efficacy of rTMS in treating cognitive impairment in AD [23]. While rTMS is a noninvasive neuromodulation technique that enhances synaptic plasticity and may slow cognitive decline, prior studies have been limited by small sample sizes and insufficient follow-up data. This study aimed to address these gaps through a randomized, double-blind, placebo-controlled clinical trial. A total of 135 participants with AD were randomly assigned to either an active rTMS group or a sham treatment group. Treatment was administered five days per week over two phases (2 weeks and 4 weeks). Cognitive function was assessed at baseline and at multiple intervals, including a 6-month follow-up. Results showed that both active and sham rTMS led to significant short-term cognitive improvements, but no sustained difference was observed between groups over the full treatment period. Adverse effects, such as headaches and dizziness, were minimal and well-tolerated. Despite these findings, rTMS remains a promising area of research, particularly when combined with cognitive training or targeted brain region stimulation. Further studies are needed to refine treatment protocols and establish long-term cognitive benefits for AD patients.

Yu et al. investigated the effects of aerobic exercise on cognitive decline in early AD, comparing cycling to stretching in a randomized trial [11]. Given inconsistent findings in previous research, the study aimed to determine whether aerobic exercise slows cognitive decline more effectively than stretching. A total of 96 participants with early AD were assigned to either a cycling or stretching group, exercising three times per week for six months. Cycling was chosen to accommodate participants with hip or knee arthritis. Cognitive function, including executive function, memory, attention, processing speed, and language, was assessed using standardized neuropsychological tests, while ADAS-cog and MMSE tracked overall cognitive decline. Exercise intensity was regulated using heart rate reserve (HRR), with a target increase over time. The mean HRR for cyclists remained at 54%, below the target range of 70–75% by the end of the study. While ADAS-cog results showed no significant differences between groups, cyclists exhibited a significant decline in attention, processing speed, and language, though memory remained unaffected. Despite the lack of a control group, comparisons with external data on natural cognitive decline suggested a potential benefit of aerobic exercise. They concluded that aerobic exercise may reduce cognitive decline, though between-group differences were not statistically significant. The absence of MRI or PET imaging limited insight into underlying neural mechanisms, highlighting the need for further research to clarify the cognitive impact of exercise in AD.

Gómez-Gallego et al. investigated the impact of music interventions on cognition and motor function in AD patients, comparing active music intervention (AMI), receptive music intervention (RMI), and a non-musical control [9]. AMI involved playing or producing music, while RMI focused on listening and discussing personal musical preferences. This quasi-experimental study included 90 patients with mild-to-moderate dementia across six nursing homes, assigned to AMI, RMI, or a control group that watched nature documentaries. Over three months, participants engaged in twice-weekly sessions featuring 12 three-minute musical pieces or videos. AMI participants followed structured musical routines, while RMI participants received personalized playlists and engaged in discussions about their song choices. Cognition and motor function were assessed using the MMSE, NPI, GDS, Barthel Index (BI), and Tinetti Scale (TS). The AMI group showed significant improvements in MMSE, BI, and TS scores, alongside a decrease in NPI scores, indicating slowed cognitive decline and preserved motor function. The RMI group exhibited similar trends without statistical significance but maintained stability compared to the control group, which declined. A key limitation was the inclusion of patients on AD-related medications, which may have influenced results. The authors suggest that further personalization of musical interventions, such as tailored playlists or individualized music creation, could enhance therapeutic benefits.

The efficacy of rTMS targeting the precuneus (PC) region was investigated as a potential treatment for AD [15]. While rTMS has shown promise in slowing cognitive decline, optimal stimulation sites remain unclear. This study aimed to evaluate PC-rTMS compared to sham stimulation over 24 weeks. A double-blind, sham-controlled trial was conducted with 50 participants diagnosed with mild to moderate AD, confirmed by MMSE, CDR-SB, and CSF biomarkers. Patients were randomized to receive either PC-rTMS or sham treatment, with cognitive and functional assessments (ADAS-cog11, ADCS-ADL, frontal assessment battery, NPI) conducted every 12 weeks. MRI was used to ensure precise coil placement for stimulation. Results indicated significant improvements in MMSE and CDR-SB scores over time in the PC-rTMS group, suggesting a stabilization of cognitive decline. Cortical activity correlated positively with CDR-SB scores, further supporting the treatment’s potential. PC-rTMS was well-tolerated, with only mild neck discomfort reported in some patients. However, due to individual differences in brain electrical fields, the technique was not personalized and may be less effective in early AD cases. These findings highlight PC-rTMS as a safe and promising intervention for AD, warranting further research to refine its application and individualize treatment approaches.

### 3.3. Critical Perspective Across New Therapeutic Strategies in AD

Across all therapeutic domains, ranging from symptomatic cholinergic modulation and immunotherapies to nanotechnology, natural products, gut–brain axis interventions, and neuromodulation, a consistent translational gap emerges between mechanistic plausibility, preclinical efficacy, and clinically meaningful disease modification. A dominant methodological limitation is the overreliance on reductionist models (enzyme assays, docking simulations, acute toxin-induced phenotypes, or short-term cognitive endpoints) that incompletely capture the temporal, cellular, and network-level complexity of AD. Even when studies incorporate multimodal validation, there remains a recurring disconnect between target engagement (such as amyloid clearance, tau reduction, receptor activation, microbiome shifts, or synaptic modulation) and durable functional or disease-modifying outcomes. This suggests that AD pathophysiology is not governed by linear, single-pathway causality but rather by dynamic, interacting systems in which compensatory mechanisms, disease stage, and inter-individual heterogeneity critically shape therapeutic response. Furthermore, across pharmacological and non-pharmacological strategies alike, insufficient integration of longitudinal biomarkers, limited standardization of intervention parameters, and underpowered or highly variable study designs collectively constrain reproducibility and cross-study comparability. Taken together, these limitations indicate that future progress will depend less on incremental optimization of isolated targets and more on the development of integrated, systems-level therapeutic frameworks that combine early-stage biomarker-guided stratification, multimodal intervention design, and rigorous validation in clinically representative models capable of capturing disease progression over time.

### 3.4. Last Point

Contemporary AD research highlights a clear shift from purely symptomatic cholinesterase inhibition toward multi-target, disease-modifying strategies that integrate pharmacological, natural, and neuromodulatory approaches. Optimized acetylcholinesterase inhibitors, improved dosing regimens, and advanced delivery systems continue to enhance cholinergic function with better tolerability, while immunotherapies have underscored the complexity of amyloid and tau pathology and the importance of early intervention guided by biomarker-based patient selection. In parallel, emerging molecular targets and nanotechnology platforms are enabling greater mechanistic precision, and adjunct approaches such as antioxidants, probiotics, and non-invasive neuromodulation are contributing supportive and potentially synergistic effects on cognition and neuroprotection. Among the approaches discussed, those targeting early-stage disease mechanisms with biomarker-guided stratification appear to hold the most consistent promise, as exemplified by amyloid-targeting immunotherapies such as lecanemab and donanemab, which demonstrate slowed cognitive decline in carefully selected early AD populations, reinforcing the critical roles of timing and disease stage. In addition, therapies aimed at neuroinflammation and microglial regulation, including TREM2 modulation and masitinib, are increasingly compelling because they address broader and more interconnected pathological networks beyond amyloid accumulation alone. Although nanotechnology-enabled delivery systems and neuromodulatory interventions remain largely exploratory or preclinical, they further expand the therapeutic landscape by improving targeting, bioavailability, and network-level brain function. Overall, current evidence most strongly supports future combination strategies that integrate early biomarker-based diagnosis with simultaneous targeting of amyloid pathology, neuroinflammation, synaptic dysfunction, and metabolic impairment, rather than relying on single-target interventions.

## 4. Parkinson’s Disease

### 4.1. Epidemiology and Pathomechanisms

PD is a progressive neurodegenerative disorder that affects over 6 million individuals globally [36]. Based on age of onset and underlying etiology, PD is classified into several subtypes: early- or late-onset, familial or sporadic, and monogenic or idiopathic forms [37]. Idiopathic PD, the most common form, lacks a known cause, whereas genetic forms result from inherited mutations in genes such as synuclein alpha (SNCA), leucine-rich repeat kinase 2 (LRRK2), and PARKIN [38]. Atypical Parkinsonian syndromes, including multiple system atrophy (MSA), progressive supranuclear palsy (PSP), and corticobasal degeneration (CBD), present with PD-like motor symptoms but are pathologically distinct [39]. Although PD predominantly affects individuals over the age of 60, approximately 5–10% of cases are classified as early-onset, occurring before age 50 [40], while the majority present after age 70. Neurodegeneration often begins years before the appearance of motor symptoms, complicating early diagnosis and the identification of causal factors [41]. PD is the second most common neurodegenerative disorder after AD, affecting 1–2% of individuals over 65 years of age worldwide [42]. The precise etiology of PD remains incompletely understood, but both genetic and environmental factors contribute to disease risk [37,43]. Familial forms of PD, which may present as early- or late-onset, are commonly associated with single-gene mutations in *SNCA*, *PINK1*, *PARK7*, *VPS35*, and *PRKN*, which encode proteins involved in α-synuclein regulation, mitochondrial function, endosomal trafficking, and oxidative stress responses [37,40]. In contrast, sporadic PD is believed to result from polygenic risk and environmental exposures, with *GBA* and *LRRK2* mutations being particularly associated with increased susceptibility [37]. Environmental risk factors include chronic exposure to pesticides, heavy metals, industrial chemicals, and repeated head trauma [44,45], while aging remains a dominant risk factor due to the progressive loss of dopaminergic neurons in the substantia nigra.

Pathologically, PD is characterized by the formation of Lewy bodies composed of misfolded α-synuclein aggregates, along with the degeneration of dopaminergic neurons in the substantia nigra, which disrupts basal ganglia signaling and impairs motor control [37,40]. These changes manifest clinically as motor symptoms such as tremors, bradykinesia, rigidity, and postural instability [36,37]. Non-motor symptoms are also prominent and result from dysfunction in multiple neurotransmitter systems: noradrenergic and serotonergic deficits contribute to depression, sleep disturbances, pain, and hyposmia, while cholinergic dysfunction is linked to cognitive decline and dementia [36,40]. Current treatment strategies primarily aim to restore dopaminergic signaling, with oral levodopa being the most effective symptomatic therapy [36]. However, this approach is limited by fluctuating dopamine levels, adverse effects, and the absence of disease-modifying or neuroprotective properties [36]. Given the rising global prevalence of PD, there is an urgent need for more effective therapeutic options that not only alleviate symptoms but also slow disease progression and protect neuronal integrity [40]. PD progresses in a staged manner, commonly assessed by the Hoehn and Yahr scale [46], which categorizes disease severity from Stage 1 (mild, unilateral symptoms) to Stage 5 (advanced, bedridden or wheelchair-bound). As the disease advances, patients experience increased motor disability and dependence in activities of daily living. Cognitive and psychiatric complications are frequent and significantly impact quality of life. Cognitive decline ranges from mild executive dysfunction to PD dementia (PDD), which impairs memory, visuospatial abilities, and problem-solving. Psychiatric symptoms such as depression, anxiety, apathy, and hallucinations are also common; depression, in particular, is closely linked to dopaminergic deficits, while hallucinations may arise from disease progression or as adverse effects of dopaminergic therapy [47]. These multifaceted clinical features underscore the complex, multisystem nature of PD and highlight the importance of comprehensive, individualized management strategies.

### 4.2. New Therapeutic Reports

**Figure 2:**
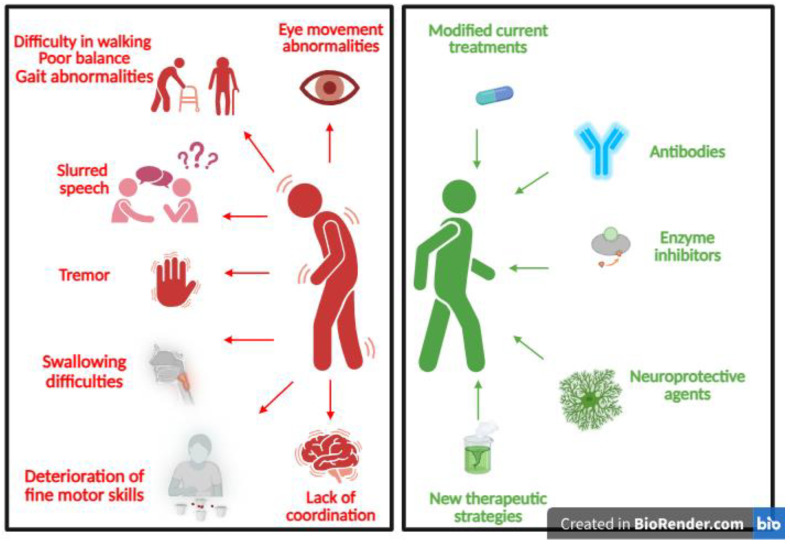
Clinical manifestations and emerging therapeutic strategies in Parkinson’s disease (PD).

Recent progress in PD research has led to the exploration of innovative therapeutic approaches aimed at improving symptom management and altering disease progression. Moving beyond conventional dopaminergic therapies, these strategies target multiple aspects of PD pathophysiology, including neuroinflammation, oxidative stress, and protein aggregation (Table 2). The following sections summarize emerging modalities such as modified current treatments, antibody-based therapies, enzyme inhibitors, neuroprotective agents, and novel therapeutic strategies under investigation (Figure 2).

#### 4.2.1. Modified Current Treatments

Foslevodopa/foscarbidopa, a novel subcutaneous (s.c.) infusion therapy composed of levodopa phosphate and carbidopa phosphate, was developed to address the motor fluctuations and “off” periods associated with oral levodopa/carbidopa (LD/CD) administration in PD. This formulation aims to provide continuous dopaminergic stimulation while minimizing the side effects linked to peak plasma dopamine levels. Preclinical pharmacokinetic studies conducted in rats, dogs, monkeys, and Göttingen minipigs demonstrated that s.c. infusion of foslevodopa/foscarbidopa achieved stable and therapeutically relevant plasma LD concentrations, as measured by HPLC-tandem MS. Subsequent clinical trials in humans evaluated optimal dose ratios, infusion protocols, and comparisons to oral LD/CD. In the group, participants were randomized to receive foslevodopa/foscarbidopa infusion, placebo, or oral LD/CD. Group 2 tested various dose ratios, while group 3 examined the effects of a loading dose followed by a 72 h continuous infusion. The 20:1 foslevodopa/foscarbidopa ratio most closely matched the pharmacokinetic profile of oral LD/CD, producing more stable plasma LD levels and reducing motor fluctuations. Notably, a loading dose enabled patients to reach steady-state LD/CD levels within ~2 h, compared to 12–16 h without a loading dose. These findings highlight the potential of foslevodopa/foscarbidopa infusion as a more consistent and controlled dopaminergic therapy for PD. However, the feasibility of widespread clinical use depends on the development of a fully portable subcutaneous infusion pump system [48].

LD/CD remains the cornerstone of PD management; however, oral administration results in fluctuating plasma levels, contributing to “off” periods, motor complications, and progressive neuroadaptations. To address these limitations, ND0612, a liquid LD/CD formulation delivered via continuous subcutaneous infusion, was evaluated for its ability to maintain more stable dopaminergic stimulation. Patients received eithe14 h24 h or a 14-h infusion, with the latter group also receiving an oral LD/CD dose in the morning to accelerate symptom control. Across both groups, treatment resulted in a ~2 h reduction in “off” time and a ~3.3 h increase in “on” time without troublesome dyskinesia. Although not designed for direct comparison, the 24 h infusion group showed additional benefits, including improvements in morning akinesia and sleep quality, potentially due to continuous overnight dopaminergic coverage. Adverse events were generally mild and consistent with the known safety profile of oral LD/CD. Local infusion site reactions were comparable to those seen with other subcutaneous delivery systems. These findings support ND0612 as a feasible and well-tolerated alternative to oral and device-assisted therapies, offering more consistent symptom control in PD [49].

Liu et al. explored the potential synergistic effects of Nardosinone, a compound derived from traditional Chinese medicine, in combination with levodopa, the gold standard treatment for PD. While levodopa remains the cornerstone of PD therapy, its long-term use often results in complications such as motor fluctuations and dyskinesia. The study utilized a rotenone-induced PD rat model to evaluate whether co-treatment with Nardosinone and levodopa could improve therapeutic outcomes. Behavioral assessments showed that rats receiving the combination therapy demonstrated significantly improved motor performance compared to those treated with levodopa alone. Histological analysis further confirmed reduced neuronal damage in the substantia nigra, indicating a neuroprotective effect of the combined treatment. A novel and significant finding of the study was the role of gut microbiota in modulating the therapeutic effects. Nardosinone was found to restore neurotransmitter levels in the gut and enhance central dopamine biosynthesis, potentially via its interaction with intestinal flora. Five key metabolites of Nardosinone were identified, suggesting that gut-mediated biotransformation may be critical for its pharmacological action. Overall, the results support the complementary use of Nardosinone with levodopa as a balanced therapeutic strategy that addresses both motor and non-motor symptoms of PD. This approach integrates principles of traditional Chinese medicine with modern pharmacotherapy. The authors recommend further research to validate these findings and to assess the long-term safety and efficacy of the combination therapy [50].

#### 4.2.2. Antibodies

The efficacy of prasinezumab, a humanized monoclonal antibody targeting aggregated α-synuclein, is being evaluated in the ongoing Phase II trial of anti-α-synuclein antibody in early Parkinson’s disease (PASADENA) trial, a 5-year multicenter study designed to assess its potential disease-modifying effects in early PD [51]. Prasinezumab is hypothesized to slow disease progression by binding to extracellular α-synuclein aggregates, thereby inhibiting their cell-to-cell transmission and mitigating neurodegeneration. Participants in the PASADENA trial were stratified into three cohorts: (1) early-start, who received prasinezumab from the outset of the study; (2) late-start, who received placebo for the first year followed by prasinezumab for the remaining duration; and (3) an external comparator group from the Parkinson’s Progression Markers Initiative (PPMI), who did not receive any study-related intervention. Clinical assessments included the Movement Disorder Society-sponsored revision of the Unified Parkinson’s Disease Rating Scale (MDS-UPDRS), cognitive evaluations, and dopamine transporter imaging with single-photon emission computed tomography (DaT-SPECT). While DaT-SPECT imaging did not reveal substantial differences between groups, patients receiving prasinezumab demonstrated a slower rate of motor and non-motor symptom progression, including a reduced incidence of postural instability, as measured by the MDS-UPDRS. Notably, during years 3 and 4 of the trial, the initiation of dopaminergic therapy was common in both PASADENA and PPMI cohorts. Improvements in patients concurrently receiving prasinezumab and dopaminergic medication suggest a potential synergistic or additive therapeutic effect. Collectively, these findings indicate that long-term treatment with prasinezumab may confer clinical benefits by decelerating the progression of early-stage PD, warranting further investigation in larger, confirmatory trials [51]. Also, a retrospective subgroup analysis suggests that prasinezumab may benefit rapidly progressing PD patients, but due to potential biases, future research should focus on improved models and biomarker-driven patient selection to validate its efficacy and safety [72].

#### 4.2.3. Enzyme Inhibitors

Mutations in the GBA gene result in reduced activity of lysosomal glucocerebrosidase (GCase), leading to the accumulation of its substrate, glucosylceramide (GL-1). This accumulation contributes to a pathological feedback loop in which elevated GL-1 levels promote the aggregation of α-synuclein, further impairing GCase function and exacerbating α-synuclein toxicity within the CNS. Venglustat, an oral glucosylceramide synthase inhibitor, is designed to disrupt this pathogenic cycle by reducing GL-1 synthesis and thereby potentially attenuating α-synuclein accumulation. The safety, tolerability, and pharmacodynamic effects of venglustat were evaluated in Part 1 of the Multicenter pharmacOkinetics and interVEntional Study in Parkinson’s Disease (MOVES-PD), a 3-year clinical trial investigating venglustat in patients with GBA-associated PD. Participants were stratified by ethnicity (Japanese and non-Japanese) and randomized to receive either placebo or one of three venglustat doses (low, medium, or high) administered orally once daily. Among Japanese participants, each group (placebo, low, medium, high) included 3 patients. In the non-Japanese cohort, the distribution included 4 patients in the placebo group, 4 in the low-dose group, 5 in the medium-dose group, and 4 in the high-dose group. Patients were monitored at multiple time points, day 2, day 3, week 2, week 4, week 8, and then every 4 weeks, throughout the study duration (52 weeks for Japanese and 36 weeks for non-Japanese participants). At each visit, safety assessments were conducted and plasma samples collected. Venglustat demonstrated an acceptable safety profile across all dosing groups and effectively reduced GL-1 levels in both plasma and CSF during the initial 4-week period, in a dose-dependent manner. Although changes in other biomarkers were not observed within this short-term analysis, the ability of venglustat to cross the BBB and modulate its biochemical target in the CNS is encouraging. These preliminary findings support further investigation into its long-term efficacy and potential disease-modifying effects in GBA-associated PD [52].

Cholinergic degeneration, particularly in the basal forebrain neocortex and mesencephalic locomotor regions, is known to contribute significantly to impairments in gait and cognition in PD, thereby increasing the risk of falls. Rivastigmine, an AChE-I, is being investigated for its potential to mitigate these deficits by enhancing central cholinergic transmission. Phase 3 of the Cholinesterase Inhibitor to Prevent Falls in Parkinson’s Disease (CHIEF-PD) trial is currently evaluating the efficacy of transdermal rivastigmine in reducing fall incidence over a 12-month period in patients with PD. Participants in the trial were randomized in a double-blind fashion to receive either rivastigmine or placebo via daily transdermal patches. The dosing regimen began at 4.6 mg/24 h and was titrated to 9.5 mg/24 h at one month, with a further increase to 13.3 mg/24 h at six months, depending on individual tolerability. Comprehensive assessments were conducted at baseline and at the 12-month endpoint. In addition to in-person evaluations, monthly telephone interviews and patient-maintained diaries were utilized to monitor fall frequency and other clinical outcomes. The CHIEF-PD trial also aims to investigate the broader effects of rivastigmine on both motor and non-motor symptoms. Outcome measures include evaluations of cognition, gait, balance, dysphagia, depression, and health-related quality of life, along with an analysis of the cost-effectiveness of rivastigmine treatment. As the trial is ongoing, final results have not yet been reported [53].

Ferroptosis, a form of iron-dependent, lipid peroxidation-driven cell death, has been increasingly implicated in the neurodegeneration observed in PD. Activation of 15-lipoxygenase (15-LO) catalyzes peroxidation of polyunsaturated fatty acids, promoting ferroptotic pathways that generate reactive oxygen species (ROS), inflammation, and lipid peroxidation, all of which contribute to α-synuclein aggregation and neuronal loss. To target this mechanism, PTC-041, a selective 15-LO inhibitor, was evaluated for its anti-ferroptotic and neuroprotective effects in both in vitro and in vivo PD models. In vitro studies using neuronal cultures derived from rodent models and PD patient samples demonstrated that PTC-041 inhibited ferroptosis, reduced α-synuclein aggregation, and improved neuronal survival. In vivo, PTC-041 was tested in two PD models: 6-hydroxydopamine (6-OHDA)-lesioned female Sprague Dawley rats and Line 61 transgenic mice. In rats, intracerebral 6-OHDA administration induced dopaminergic degeneration, and PTC-041 treatment resulted in improved motor function, as assessed by cylinder and apomorphine-induced rotation tests. In Line 61 mice, treatment over 6 or 15 weeks led to reduced α-synuclein pathology and preservation of neuronal markers (NeuN, MAP2, Glut1), as confirmed by immunofluorescent analysis. These findings support the potential of PTC-041 as a disease-modifying agent that attenuates ferroptotic cell death and ameliorates both pathological and functional deficits in PD models [54].

#### 4.2.4. Neuroprotective Agents

(-)-Clausenamide (Clau) was evaluated for its potential neuroprotective effects on dopaminergic neurons in PD, addressing the current lack of therapies that mitigate oxidative stress-induced neuronal degeneration. Given previous evidence of Clau’s antioxidant properties, this study assessed its efficacy and mechanisms of action in both 1-Methyl-4-phenyl-1,2,3,6-tetrahydropyridine (MPTP)-induced PD mouse models and in vitro neuronal cultures. Mice were treated with Clau (low or high dose), ferrostatin-1 (Fer-1), selegiline, or saline, and compared with untreated MPTP and healthy controls. Behavioral assays revealed that Clau, Fer-1, and selegiline significantly improved motor performance, including gait, climbing, and fall latency. Biochemical analyses showed restoration of dopamine, dihydroxypheny-phenylacetic acid, and Homovanillic acid levels, as well as attenuation of dopaminergic neuronal loss. Lipid peroxidation markers malondialdehyde (MDA) and 4-hydroxynonenal (4-HNE), associated with ferroptosis, were reduced by Clau in a dose-dependent manner, indicating anti-ferroptotic activity. In vitro, primary neuronal cultures derived from neonatal mice demonstrated that Clau inhibited Protein kinase C α (PKCα)-mediated activation of ALOX5 by forming a hydrogen bond at Ser663, thereby preventing lipid peroxidation. Together, these findings support Clau as a promising neuroprotective agent in PD through suppression of ferroptosis-mediated lipid peroxidation, meriting further preclinical and translational investigation [55].

Histamine signaling has been implicated in PD pathogenesis, prompting investigation into the therapeutic potential of antihistamines. Analysis of National Health Insurance Service (NHIS) data identified fexofenadine as a candidate drug associated with slower PD progression. To evaluate its efficacy, fexofenadine was tested in two PD mouse models: 6-hydroxydopamine (6-OHDA)-lesioned mice and α-synuclein preformed fibril (PFF)-injected M83 transgenic mice. Mice in both models received either vehicle or oral fexofenadine (20 mg/kg/day) for 4 weeks. Behavioral assessments and immunohistochemical analysis revealed that fexofenadine improved motor performance and reduced microgliosis in 6-OHDA mice. In the PFF model, treatment significantly reduced α-synuclein aggregation and neuroinflammation. These effects were supported by bimolecular fluorescence complementation transgenic *C. elegans*, where fexofenadine limited α-synuclein accumulation. Bulk RNA sequencing of brain tissue from PFF mice showed that fexofenadine normalized the expression of inflammation- and immune-related pathways upregulated by PFF injection. Collectively, these findings suggest that fexofenadine exerts neuroprotective effects by modulating microglial activation and inflammatory signaling, supporting its potential as a repurposed therapeutic for PD [56].

Gut microbiota dysbiosis is commonly observed in PD, with increased Lactobacillus and Enterobacteriaceae and decreased Prevotella, *Clostridium coccoides*, and *Bacteroides fragilis*. Given the link between intestinal dysregulation and PD progression, ceftriaxone, a β-lactam antibiotic with known neuroprotective properties, was investigated in a PD mouse model. Six groups were assessed: controls (saline-treated), MPTP-only (M), MPTP plus ceftriaxone (MCEF), MPTP plus ceftriaxone and fecal transplant from PD mice (MCEFF), fecal transplant alone (FMT), and fecal transplant followed by ceftriaxone (FCEF). Behavioral testing, immunofluorescence, western blotting, cytokine profiling, and 16S rDNA sequencing were performed. Ceftriaxone-treated mice (MCEF, FCEF) showed improved motor function, reduced microglial activation in the substantia nigra, and restored expression of brain-derived neurotrophic factor (BDNF) and glial cell line-derived neurotrophic factor (GDNF), in contrast to MPTP-only and FMT groups. Furthermore, intestinal inflammation was reduced, and tight junction protein expression was elevated in ceftriaxone-treated mice, indicating improved gut barrier integrity. These results support ceftriaxone as a potential neuroprotective treatment for PD, via modulation of neuroinflammation and gut–brain axis signaling [57].

Ginkgo biloba extract (GB) and Protocatechuic acid (PCA), both widely used in traditional Chinese medicine, have demonstrated neuroprotective properties and were evaluated for their potential therapeutic effects in PD. In in vitro studies, PC12 cells were seeded into well plates and exposed to rotenone to induce mitochondrial dysfunction and neuronal injury. Cells were subsequently treated with GB, PCA, or a combination of both agents. Analysis revealed that all treatment groups exhibited increased cell viability and reduced cytotoxicity, with the combination treatment showing the most pronounced effects, suggesting a synergistic neuroprotective action. Further biochemical assays demonstrated that GB and PCA treatments significantly reduced ROS levels while enhancing antioxidant defenses, as evidenced by elevated activity of glutathione (GSH), superoxide dismutase (SOD), and catalase. These protective effects were then validated in vivo using an MPTP-induced mouse model of PD. Experimental groups included non-induced controls and MPTP-induced mice treated with madopar (levodopa/benserazide), GB, PCA, or the GB-PCA combination. Behavioral testing indicated that mice receiving the combined GB and PCA treatment demonstrated the most significant improvements in motor coordination. Immunohistochemical staining of brain sections showed an increased number of neurons in the substantia nigra and a higher count of TH^+^ neurons in the midbrain compared to untreated MPTP mice. Furthermore, the combination therapy most effectively restored antioxidant enzyme activity (SOD, catalase, GSH) and mitigated oxidative stress in brain tissue. In summary, the combined administration of GB and PCA confers synergistic neuroprotection by reducing oxidative damage, preserving dopaminergic neurons, and enhancing motor functions in PD models. These findings support the potential for combined phytotherapeutic strategies in PD treatment and warrant further preclinical and clinical investigation [58].

Takeshige-Amano et al. investigated the potential neuroprotective effects of selegiline on white matter (WM) microstructures in patients with PD. Selegiline, a monoamine oxidase B (MAO-B) inhibitor, has been widely used to enhance dopaminergic signaling, but its effects on WM integrity have not been fully elucidated. The study employed diffusion tensor imaging and free-water (FW) imaging to assess WM changes and distinguish neuroinflammation from neurodegeneration in the substantia nigra and major WM tracts. The analysis included PD patients treated with selegiline, PD patients not receiving selegiline, and healthy controls, using imaging data from two independent cohorts to ensure robustness and reproducibility. The findings revealed that untreated PD patients exhibited significant microstructural abnormalities in multiple WM tracts. In contrast, selegiline-treated patients demonstrated fewer abnormalities and reduced free-water levels in key WM regions such as the inferior fronto-occipital fasciculus and superior longitudinal fasciculus, which are involved in motor control and cognitive function. Notably, in several regions, no significant differences were observed between selegiline-treated patients and healthy controls, suggesting that selegiline may help preserve WM integrity. Despite the promising results, the authors acknowledged limitations, including a relatively small sample size and the retrospective design of the study. They concluded that further prospective, longitudinal studies are warranted to validate the neuroprotective role of selegiline and to inform personalized treatment strategies for PD [59].

Shang et al. investigated the role of the gut microbiota in the absorption and metabolism of FLZ, a novel neuroprotective compound currently in clinical trials for PD. FLZ has demonstrated promising anti-inflammatory and neuroprotective effects in preclinical models. However, the pharmacokinetic mechanisms underlying their bioavailability remained unclear prior to this study. Using a combination of in vitro and in vivo experiments, the authors identified that FLZ is primarily metabolized by gut microbial lanosterol 14-alpha-demethylase into M1, a major metabolite. M1 is then efficiently absorbed into the bloodstream and converted back to FLZ by catechol O-methyltransferase (COMT) in the systemic circulation, enabling its therapeutic activity in the CNS. Importantly, the study demonstrated that disruption of gut microbiota, such as through antibiotic treatment, significantly impaired FLZ metabolism and absorption. In MPTP-induced PD mouse models, antibiotic-induced dysbiosis led to reduced FLZ bioavailability and diminished neuroprotective effects, highlighting the essential role of a functional and balanced gut microbiome for optimal drug efficacy. Additionally, the findings suggest that the neuroprotective, immunomodulatory, and gut microbiota-stabilizing properties of FLZ (referred to as C423 in some contexts) may be interdependent, further reinforcing the concept of the gut–brain axis in PD pathophysiology and treatment response. The authors also caution against concurrent use of broad-spectrum antibiotics with microbiota-dependent therapeutics such as FLZ, due to the risk of reduced therapeutic benefit [60].

Knez et al. investigated the therapeutic potential of azastilbene-based compounds as selective MAO-B inhibitors for the treatment of PD. MAO-B inhibitors have garnered attention for their neuroprotective and symptomatic benefits. However, existing MAO-B inhibitors often suffer from off-target effects and suboptimal selectivity, limiting their clinical utility. In this study, the authors screened a library of azastilbene derivatives, identifying lead compounds with high potency and MAO-B selectivity. These candidates underwent structural optimization to enhance specificity and reduce non-selective hydrophobic interactions, which can lead to adverse effects. Molecular docking studies provided detailed insights into the binding affinity and selectivity of the optimized compounds for the MAO-B active site. Functional validation in cellular PD models showed that the azastilbene-based inhibitors mitigated rotenone-induced oxidative stress. Further, *vivo* testing in an MPTP-induced PD mouse model confirmed that the compounds could alleviate motor impairments and protect dopaminergic neurons, indicating a robust neuroprotective effect. The findings support azastilbene derivatives as promising next-generation MAO-B inhibitors, potentially offering superior efficacy and safety profiles compared to current drugs such as selegiline or rasagiline. The authors note, however, that comprehensive studies on pharmacokinetics, bioavailability, and long-term safety are required before clinical translation. If successfully developed, these compounds could contribute to disease-modifying therapies for PD, addressing both motor symptoms and underlying neurodegeneration [61].

Cao et al. investigated benzothiazole derivatives for PD treatment, focusing on MAO-B inhibition, antioxidant, and anti-inflammatory effects. Compound 3h showed potent and selective MAO-B inhibition (IC50 = 0.062 µM) with competitive and reversible binding, strong antioxidant capacity, and metal-chelating abilities against Fe^2+^ and Cu^2+^, which are involved in oxidative stress. Molecular docking revealed key interactions with MAO-B active site residues. In cellular models, 3h protected PC-12 cells from oxidative damage and reduced pro-inflammatory markers like TNF-α, nitric oxide (NO), and ROS in microglia. Importantly, it demonstrated the ability to cross the BBB, making it a promising multi-target candidate. While these results are encouraging, further in vivo studies are needed to validate its long-term efficacy and clinical potential for PD therapy [62].

#### 4.2.5. New Therapeutic Strategies

Sun et al. investigated the therapeutic potential of ursolic acid (UA) in PD, emphasizing its dual role in modulating apoptosis and neuroinflammation, two key pathological processes involved in the progressive loss of dopaminergic neurons. Targeting these interconnected mechanisms may offer disease-modifying benefits in PD. The researchers employed an integrated approach combining network pharmacology, molecular docking, and experimental validation. Bioinformatics analyses identified nine key target genes (including Caspase-3, Caspase-8, IL6, MAPK3, among others) that UA could modulate. Molecular docking demonstrated strong binding affinities between UA and apoptosis-related proteins (Caspase-3, Caspase-8) as well as inflammatory cytokines (TNF-α, IL-1β). In vivo experiments using MPTP-induced PD mouse models revealed that UA administration improved motor function and enhanced tyrosine hydroxylase (TH) expression, indicating preservation of dopaminergic neurons. In vitro studies in SH-SY5Y, a human-derived neuroblastoma cell line neuroblastoma cells, corroborated these findings, showing that UA suppressed pro-inflammatory cytokine expression (IL-6, TNF-α, IL-1β) and downregulated apoptotic proteins (Caspase-3, Caspase-8, MAPK3). Despite these promising effects, the clinical translation of UA faces challenges due to its poor bioavailability, stemming from low solubility and limited gastrointestinal (GI) absorption. The authors suggest that nanoparticle-based delivery systems, prodrug strategies, or structural modification could improve UA’s pharmacokinetic profile. While further preclinical optimization and human clinical trials are warranted, UA represents a compelling candidate for the development of novel, disease-modifying therapies in PD and potentially other neurodegenerative disorders [63].

Dai et al. developed phosphorous dendrimer-based nanocomplexes (123/FN NCs) for delivering fibronectin (FN) to the brain as a novel therapeutic strategy for PD. These nanocomplexes (~223 nm) effectively crossed the BBB and accumulated in activated microglia, where they shifted microglia from a pro-inflammatory M1 state to an anti-inflammatory M2 phenotype by reducing NF-κB signaling and lowering levels of cytokines such as IL-6, IL-1β, and TNF-α. In an MPTP-induced PD mouse model, the treatment improved motor function, preserved dopaminergic neurons, and reduced α-synuclein aggregation, highlighting its neuroprotective potential. While the results demonstrate promise for targeted drug delivery and inflammation control in PD, further studies are needed to evaluate long-term safety, pharmacokinetics, and clinical translation before this approach can be considered for human use [64].

Alimohammadi et al. investigated graphene-based nanoparticles (GNPs) for their ability to inhibit α-synuclein amyloid fibrillation, a hallmark of PD pathology. Through molecular dynamics simulations of four graphene variants, pure graphene, nitrogen-doped (N-Graphene), phosphorus-doped, and bromine and nitrogen co-doped graphene, they found that all hindered fibril formation, with N-Graphene being the most effective by destabilizing α-synuclein structure, reducing fibril compactness, and enhancing interactions with water molecules. N-Graphene also exhibited the strongest binding energy with amyloid proteins, making it a promising candidate to prevent toxic α-synuclein aggregation. This study provides a molecular basis for graphene’s potential neuroprotective effects in PD, though further experimental validation in biological models and clinical settings is needed to assess efficacy, safety, and long-term stability [65].

BIIB122 (DNL151), a brain-penetrant inhibitor of leucine-rich repeat kinase 2 (LRRK2), represents a precision medicine approach targeting genetic forms of PD. Current phase 2 clinical trials (LUMA study) are evaluating its ability to slow disease progression in early-stage PD, particularly in patients carrying LRRK2 mutations. The therapeutic rationale is based on the role of LRRK2 in regulating lysosomal function, inflammation, and neuronal survival. Early trial data indicate favorable pharmacodynamic effects and target engagement, although efficacy outcomes are still under investigation. This approach exemplifies the shift toward genotype-driven therapies and disease-modifying strategies targeting intracellular signaling pathways [66].

#### 4.2.6. Future Directions Using Genomic Evaluations

Disulfidptosis, a newly characterized form of cell death resulting from the accumulation of disulfide bonds due to redox imbalance, may play a role in PD. To investigate the relevance of disulfidptosis-related genes (DRGs) in PD, differentially expressed genes (DEGs) were extracted from the Gene4PD database and compared with known DRGs. The overlap, termed differential expression disulfidpotosis-related genes (DEDRGs), was further analyzed using receiver operating characteristic (ROC) curves and PD datasets from GEO to evaluate their diagnostic potential. Expression of candidate DEDRGs was validated via Western blotting in MPTP-induced PD mouse models versus saline-treated controls. Additionally, pan-cancer expression profiles and prognostic associations were examined using the UCSC database. Pathway enrichment and tissue-specific expression analyses were conducted to determine functional roles. Key DEDRGs identified included ACTB, ACTN4, INF2, and MYL6. In the PD model, ACTB was downregulated, while ACTN4, INF2, and MYL6 were upregulated. These genes are implicated in NF-κB signaling, tight junction integrity, apoptosis regulation, and mitochondrial function. ROC analysis indicated strong diagnostic performance and pan-cancer data showed significant dysregulation of these genes in several tumor types. These findings suggest that ACTB, ACTN4, INF2, and MYL6 may serve as novel biomarkers and therapeutic targets for both PD and cancer, linking disulfidptosis to neurodegeneration and tumor biology [67].

Genome-wide association studies (GWAS) have enabled the identification of single nucleotide polymorphisms (SNPs) associated with the development of PD. To prioritize potential therapeutic targets, Mendelian randomization (MR) was employed to evaluate the causal relationship between SNPs and gene expression, focusing on druggable genes. Expression quantitative trait loci (eQTLs) were compiled from GWAS datasets, and SNPs associated with PD were obtained from the International Parkinson’s Disease Genomics Consortium (IPDGC). MR analysis revealed 31 genes in blood and brain tissue significantly associated with PD risk. These findings were validated in an independent PD case–control cohort, where 15 genes were successfully replicated. Among them, CHRNB1, NDUFAF2, and VKORC1 were identified as targets of drugs that are either approved or in clinical development for PD. Within the replicated gene set, CD38, CTSB, CPNMB, and MAP3K12 showed the strongest associations with PD based on MR estimates. Additional MR analysis using GWAS data on PD age of onset identified BST1, CD38, CTSB, and MMRN1 as potential targets for preventative therapies. Because most therapeutic agents act on proteins rather than gene expression, protein QTLs (pQTLs) were examined for BST1, CD38, CTSB, CPNMB, and LGALS3. Of these, only CTSB and LGALS3 showed consistent direction of effect across both eQTL and pQTL datasets and were repeatedly associated with PD risk. In conclusion, the integrative MR analysis highlighted CTSB, GPNMB, CD38, RDH, IRAK3, and LMAN1 as promising novel therapeutic targets for PD. Furthermore, CHRNB1, NDUFAF2, and VKORC1, which are linked to existing or investigational drugs, were proposed for further investigation in PD treatment development [68].

Olfactory dysfunction is a common early symptom of both AD and PD. To investigate potential molecular links between these neurodegenerative diseases, overlapping DEGs were identified using datasets from the Gene Expression Omnibus (GEO). GO and Kyoto Encyclopedia of Genes and Genomes (KEGG) analyses were conducted to predict associated biological processes and pathways, while STRING was used to explore protein–protein interactions and identify hub genes. For in vivo validation, AβPP/PS1 transgenic mice were used as an AD model and A53T transgenic mice as a PD model. Olfactory function was evaluated through behavioral olfactory tests. Tissue samples were collected from the hippocampus, substantia nigra, olfactory bulb, and olfactory epithelium in 6-month-old AD, PD, and control mice. Gene expression levels were assessed via RNA extraction and RT-qPCR. Bioinformatics analysis revealed that overlapping DEGs were primarily involved in synaptic signaling and synaptic dysfunction. Five key hub genes were identified: synaptosomal-associated protein 25 (SNAP25), synapsin 1 (SYN1), synaptotagmin 1 (SYT1), growth-associated protein 43 (GAP43), and SNAP91. RT-qPCR validation confirmed altered expression of all five genes, with SNAP25 showing the most significant changes. Tissue-specific expression patterns indicated that SNAP25 and SNAP91 were markedly downregulated in the hippocampus of AD mice, whereas SNAP25 and GAP43 were significantly altered in the substantia nigra of PD mice. Notably, SNAP25 was consistently downregulated in the olfactory bulb and epithelium in both AD and PD models, correlating with impaired olfactory behavior, specifically, a reduced preference for novel scents. Finally, virtual screening of SNAP25 identified it as a promising early diagnostic and therapeutic target for both AD and PD due to its central role in synaptic function and consistent downregulation in olfactory-related brain regions [69].

Parthanatos-associated apoptosis-inducing factor (AIF) nuclease (PAAN), also known as macrophage migration inhibitory factor (MIF), plays a critical role in the parthanatos cell death pathway and may contribute to α-synuclein-mediated neurodegeneration in PD. To investigate the role of the PAAN/MIF pathway, wild-type (WT) and PAAN/MIF knockout (KO) mice were stereotactically injected with recombinant α-synuclein pre-formed fibrils (PFFs) into the striatum. Six months post-injection, WT mice exhibited a ~50% loss of dopaminergic neurons, while KO mice were protected from neuronal loss. Correspondingly, motor deficits developed in WT mice were absent in KO mice, suggesting a neuroprotective effect from PAAN/MIF deletion. To determine the functional requirement of PAAN/MIF nuclease activity, PAAN/MIF KO mice were transfected with either WT PAAN/MIF, the E22Q mutant (deficient in nuclease activity), or the P2G mutant (deficient in tautomerase activity) using adeno-associated virus (AAV) vectors. While reintroduction of WT PAAN/MIF restored susceptibility to neurodegeneration, mice expressing the E22Q mutant were resistant, indicating that nuclease activity, but not tautomerase activity, is essential for α-synuclein-induced pathology. In vitro, neuron cultures derived from WT and PAAN/MIF KO mice were treated with ABT-888, α poly (ADP-ribose) polymerase (PARP) inhibitor, to evaluate nuclear translocation of AIF and MIF in response to α-synuclein exposure. KO cultures were also transduced with lentiviruses expressing PAAN/MIF WT, E22Q, or P2G. Results demonstrated that nuclear translocation of AIF and MIF, along with PAAN/MIF nuclease activity, were required for DNA binding and cleavage during α-synuclein PFF-induced cell death. Given the absence of specific PAAN/MIF nuclease inhibitors, structure-based drug design and screening were conducted, leading to the discovery of Parthanatos-Associated Nuclease Inhibitor-1 (PAANIB-1). In WT mice injected with α-synuclein PFFs and transfected with human α-synuclein via AAV in the substantia nigra, PAANIB-1 administration conferred neuroprotection and mitigated behavioral deficits. Similar neuroprotective effects were observed in the MPTP-induced PD mouse model treated with PAANIB-1. Collectively, these findings establish PAAN/MIF nuclease as a crucial mediator of parthanatos-driven dopaminergic neurodegeneration and identify PAANIB-1 as a promising therapeutic candidate. Further studies are warranted to validate the efficacy and safety of PAANIB-1 and to advance it toward clinical application in PD treatment [70].

To identify potential therapeutic targets and repurposable drugs for PD, PD-related genes were curated from multiple databases including KEGG, OMIM, HGMD, Genotype, and DISEASE. Gene and protein expression profiles from PD patients were compared to healthy controls to assess differential expression. Protein–protein interaction (PPI) networks were constructed using data from STRING, PINA, and HuRI, and pathway enrichment analyses were performed using GO annotations and KEGG pathway mapping. To explore potential pharmacological interventions, Drug Bank was employed to identify known drug-target interactions. Distances between PD-related genes and existing drug targets were computationally assessed within the PPI networks, allowing the identification of DEGs with potential for drug targeting. Many PD-associated genes were found to be involved in critical cellular processes such as oxidative phosphorylation, energy metabolism, and ion transport, all of which are commonly disrupted in PD pathology. From this analysis, 10 existing drugs emerged as candidates for PD repurposing, although some lacked clear mechanistic links. Notably, carfilzomib and afatinib, which target the epidermal growth factor receptor (EGFR), and vorinostat, an anti-neoplastic histone deacetylase inhibitor, showed more promising therapeutic relevance. In parallel, two distinct clusters of novel gene targets were identified. The first cluster included PSMB10, SLC47A2, HDAC8, and BCL2, while the second consisted of KIT, TXNRD1, JUN, AKT1, and PML. Several of these genes are implicated in the regulation of α-synuclein aggregation, dopaminergic neuron survival, and neurodegenerative processes. Pathway analyses revealed that these targets participate in signaling cascades such as apoptosis, neurotrophin signaling, estrogen signaling, MAPK, and PI3K-Akt pathways, key mechanisms involved in neuronal maintenance and PD progression. These findings provide a robust framework for the rational selection of drug targets and repurposing of existing therapeutics for PD, highlighting the need for preclinical validation and mechanistic studies of the identified genes and compounds [71].

### 4.3. Critical Perspective Across New Therapeutic Strategies in PD

Across the diverse therapeutic strategies reviewed in PD, including modified dopaminergic delivery systems, immunotherapies, enzyme and metabolic inhibitors, neuroprotective agents, gut–brain axis interventions, and emerging genomic approaches, a consistent translational pattern emerges in which strong mechanistic rationale and preclinical efficacy frequently fail to converge into robust, durable clinical disease modification. A central limitation across studies is the heavy reliance on short-term behavioral outcomes, toxin-induced or genetic animal models, and molecular surrogate endpoints (e.g., α-synuclein aggregation, inflammatory markers, enzyme activity, or pathway enrichment), which incompletely reflect the progressive, multisystem, and compensatory nature of human PD. Even interventions demonstrating clear target engagement—such as α-synuclein antibodies, LRRK2 inhibition, ferroptosis modulation, or gut microbiome–dependent pharmacokinetics—often show dissociation between biomarker modulation and clinically meaningful motor or non-motor improvement, highlighting an unresolved mechanistic gap between pathway correction and network-level neurodegeneration. Moreover, substantial heterogeneity in patient stratification, disease stage, dosing paradigms, and outcome measures further limits cross-study comparability and obscures true effect sizes. Emerging genomic and systems biology approaches identify promising novel targets and repurposable drugs, yet these remain largely inferential, requiring extensive functional validation before translation. Collectively, these limitations suggest that PD is unlikely to be effectively addressed through single-pathway interventions alone; instead, future therapeutic success will depend on integrated strategies that combine early biomarker-driven stratification, circuit-level and systems neuroscience-informed targeting, and longitudinal clinical validation capable of capturing disease progression beyond symptomatic fluctuations.

### 4.4. Last Point

Current therapeutic approaches for PD remain largely symptomatic, with dopaminergic replacement therapies providing substantial motor relief but offering limited neuroprotection and often contributing to long-term complications such as motor fluctuations and dyskinesia. This limitation has driven a strong shift toward the development of disease-modifying strategies aimed at slowing or halting neurodegeneration by targeting core pathogenic processes, including α-synuclein aggregation, oxidative stress, mitochondrial dysfunction, neuroinflammation, and programmed cell death pathways. Emerging pharmacological and technological innovations reflect this transition, including multi-target agents such as ursolic acid that simultaneously modulate apoptotic and inflammatory signaling, selective MAO-B inhibitors that reduce oxidative burden, and advanced nanotechnology-based delivery systems designed to enhance blood–brain barrier penetration and enable targeted modulation of neuroinflammatory cells such as microglia. In parallel, drug repurposing efforts involving antihistamines and antibiotics, along with bioactive compounds derived from traditional medicine, have demonstrated potential to attenuate neuroinflammation and oxidative injury, suggesting their utility as adjunctive or combinatorial therapies with improved safety profiles. Furthermore, precision medicine approaches, including genomic profiling, biomarker-guided stratification, and real-time pharmacokinetic monitoring, are increasingly being integrated to optimize therapeutic response and minimize inter-individual variability in drug efficacy and adverse effects. Despite these advances, major translational challenges persist, particularly with regard to poor bioavailability, limited brain penetration, safety concerns, and the lack of large-scale, long-term clinical validation. Additionally, growing evidence implicating the gut–brain axis in PD pathophysiology introduces a complementary therapeutic dimension, where microbiome-targeted interventions may enhance or synergize with conventional pharmacological strategies. Overall, the field is moving toward a more integrated and multidisciplinary treatment paradigm that combines neuroprotection, symptom control, and individualized therapy design; however, substantial collaborative efforts across basic research, clinical investigation, and pharmaceutical development will be required to overcome current barriers in drug delivery, multi-target optimization, and clinical translation.

Across the broader therapeutic landscape, the most consistently promising disease-modifying strategies appear to converge on upstream and biologically central drivers of neurodegeneration rather than isolated symptomatic pathways. In particular, interventions targeting α-synuclein pathology, such as immunotherapies (such as prasinezumab) and aggregation-modulating nanotechnologies, demonstrate relatively strong mechanistic rationale and emerging clinical signals, even if overall effect sizes remain modest. Similarly, approaches aimed at restoring lysosomal and metabolic homeostasis, including modulation of the GCase–GL-1 axis (such as venglustat), and therapies targeting neuroinflammation and microglial dysfunction (like fexofenadine, ceftriaxone, and fibronectin-based nanocomplexes) show recurring support across preclinical and early translational studies. Additional mechanistically compelling strategies include LRRK2 inhibition (such as BIIB122), which directly addresses genetically validated disease drivers, and ferroptosis-targeting interventions (like PTC-041), which act on conserved pathways of oxidative and iron-dependent neuronal vulnerability. In contrast, many phytochemical-based and adjunct neuroprotective approaches, while often demonstrating robust preclinical effects, lack consistent mechanistic specificity or clinical confirmation, limiting their current translational reliability. Collectively, although no single pathway has yet achieved definitive disease modification, α-synuclein–centric, lysosomal–metabolic, and neuroinflammatory targets currently represent the most coherent and repeatedly validated directions for future therapeutic development in PD.

## 5. Amyotrophic Lateral Sclerosis

### 5.1. Epidemiology and Pathomechanisms

Motor neurons (MNs) are a distinct class of neurons that connect to peripheral muscles and regulate both involuntary and voluntary movements [73]. ALS is a progressive neurodegenerative disorder characterized by degeneration of upper and lower MNs, leading to muscle weakness, atrophy, and ultimately respiratory failure. It primarily affects voluntary muscle control and is typically fatal within 3–5 years of symptom onset [74]. ALS is a heterogeneous, fatal neurodegenerative disorder driven by genetic mutations, excitotoxicity, protein misfolding, oxidative stress, mitochondrial dysfunction, and neuroinflammation. Ongoing research explores gene therapy, stem cell interventions, and novel pharmacologic approaches to slow disease progression and improve patient outcomes [75]. ALS is classified into sporadic ALS (sALS), accounting for approximately 90–95% of cases, and familial ALS (fALS), comprising 5–10% of cases with mutations in *SOD1*, *TDP-43*, *FUS*, and *C9ORF72* [74]. The global prevalence of ALS is estimated at 2–5 per 100,000 individuals [76]. Mutations in ALS-associated genes (*SOD1*, *TDP-43*, *FUS*, *C9ORF72*) are major contributors, particularly in familial cases, though they may also appear sporadically and influence disease progression [76]. Environmental and cellular factors, including neuroinflammation, oxidative stress, and mitochondrial dysfunction, also contribute to MN damage [75,77]. Multiple interconnected mechanisms drive ALS pathogenesis, including the accumulation and aggregation of misfolded proteins such as TDP-43, increased ROSs, impaired mitochondrial function, activation of microglia and astrocytes releasing inflammatory mediators, glutamate excitotoxicity, and disruptions in RNA metabolism and protein quality control [74,75,77,78].

Diagnosis is based on clinical evaluation, detailed medical history, and neurological examination, complemented by electromyography and nerve conduction studies. Early symptoms include muscle twitching, weakness, and impaired fine motor skills, which progress to dysphagia, dysarthria, and respiratory compromise. The disease typically begins focally in either a limb (spinal onset) or bulbar region and spreads over time, with respiratory failure being the most common cause of death [74]. ALS exhibits considerable phenotypic variability, including Progressive Muscular Atrophy (PMA), characterized by predominant lower MN involvement; bulbar-onset ALS, initially affecting speech and swallowing; and ALS with frontotemporal dementia (ALS-FTD), present in approximately 15% of patients, while up to 50% exhibit some degree of cognitive impairment [77,79,80].

Adult-onset ALS is most commonly diagnosed between 55 and 75 years of age, with a mean onset of approximately 61.8 years. Juvenile (<25 years) and young-onset (<45 years) ALS are rare, accounting for 1% and 10% of cases, respectively. ALS shows a slight male predominance (male-to-female ratio 1.4:1), and incidence rates vary globally from 1.8 to 2.8 per 100,000 per year, with higher prevalence in Europe and North America [81,82]. Genetic factors, particularly *C9ORF72*, *SOD1*, *TARDBP*, and *FUS* mutations, represent major contributors, while environmental risks such as pesticide exposure, military service, smoking, and intense physical activity have been proposed but remain inconclusive [82,83].

Currently, there is no cure for ALS, and available treatments are primarily supportive. Riluzole, a glutamate receptor antagonist, modestly slows disease progression [80], while edaravone, a free radical scavenger, demonstrates benefit in selected patients [80,84]. Stem cell therapy holds promise for neuroprotection and MN replacement, with encouraging preclinical data and early-phase clinical trials [80]. Multidisciplinary supportive care, including physical, speech, and respiratory therapy, remains essential for optimizing patient outcomes and quality of life [75,85].

### 5.2. New Therapeutic Reports

Over the years, the search for effective therapeutics in ALS has evolved significantly, with growing emphasis on neuroprotective drugs, gene-targeted therapies, pharmaceutical interventions, and stem cell-based approaches. Several recent studies have investigated novel molecular targets and therapeutic compounds aimed at modulating key pathological mechanisms implicated in ALS, including oxidative stress, neuroinflammation, hormonal imbalance, and protein aggregation. In Table 3, the novel treatments are organized under an alternative categorization, with concise descriptions of each study’s main features.

#### 5.2.1. Oxidative Stress & Neuroprotective Modulation

Multitarget hybrid derivatives of fasudil were designed to combine ROCK inhibition with NRF2 activation, targeting key mechanisms in ALS such as oxidative stress and neuroinflammation. These hybrids incorporate antioxidant moieties from ferulic and caffeic acids and were synthesized using microwave-assisted methods. Their biological activity was evaluated through kinase assays, luciferase reporter assays, qPCR, and studies in multiple cell lines, including KEAP1-deficient models to clarify the mechanism of NRF2 activation. Among the compounds, derivative 1d demonstrated the most promising multitarget profile, significantly upregulating NRF2-regulated genes and promoting NRF2 activation. Importantly, it showed strong efficacy in lymphoblastoid cells derived from SOD1-ALS patients, while having minimal effects in sporadic ALS cells. This selective response highlights its potential as a genotype-specific therapeutic candidate. Overall, compound 1d emerges as a strong lead for ALS treatment, warranting further optimization of pharmacokinetic and safety properties for future clinical development [76].

The safety, tolerability, and preliminary efficacy of fasudil, a ROCK inhibitor, were investigated in a phase 2, randomized, double-blind, placebo-controlled trial across 19 ALS centers in Germany, France, and Switzerland [86]. Participants aged 18–80 years meeting revised El Escorial criteria received either 30 mg or 60 mg IV fasudil twice daily or placebo for 20 days over 4 weeks. Co-primary endpoints were safety and tolerability through day 180, with ALSFRS-R, Motor Unit Number Index (MUNIX), Slow Vital Capacity (SVC), and survival time as secondary outcomes. Pharmacokinetic analyses measured hydroxyfasudil levels in CSF. Fasudil was well tolerated, with no serious drug-related adverse events. While ALSFRS-R scores did not differ significantly from placebo, MUNIX indicated possible early therapeutic signals at days 26 and 90. A sex-specific effect was noted, as female patients receiving 60 mg fasudil exhibited slower SVC decline. These results confirm fasudil’s safety and suggest that longer treatment durations and higher doses may be required for efficacy. Future trials should consider oral formulations to improve compliance and extended follow-up for disease-modifying effects [86].

Lee et al. explored a novel combinational therapy comprising Nebivolol and Donepezil for ALS treatment. Leveraging AI-based drug screening, the study identified this combination as synergistically targeting neuroinflammation and neuronal loss. In vitro studies utilized BV-2 microglial cells, while in vivo efficacy was tested in SOD1^G93A^ mice. Techniques included qRT-PCR, neuroinflammation and neurogenesis assays, muscle atrophy evaluation, and behavioral and survival analyses. The combination reduced proinflammatory cytokines via inhibition of NF-κB nuclear translocation, and activated the PI3K-Akt pathway, conferring neuroprotection. Treated mice demonstrated improved MN survival and extended lifespan. Despite promising results, limitations include model specificity (familial rather than sporadic ALS). Future clinical trials are required to evaluate human safety, pharmacokinetics, and efficacy [91].

The efficacy of oral tauroursodeoxycholic acid (TUDCA) in ALS patients is investigated using data from the Emilia Romagna Regional ALS Registry (Italy). That retrospective cohort included 86 treated and 172 untreated patients. High-dose TUDCA significantly prolonged survival (56.5 vs. 36.2 months) without major adverse effects. Mild GI disturbances and dermatologic reactions were reported in 20.9% of patients, with only 8.1% discontinuing therapy. ALSFRS-R scores remained stable, indicating slow disease progression. These findings support high-dose TUDCA as a safe adjunctive therapy, particularly in spinal-onset ALS with slow progression. Further prospective, randomized trials are needed to validate survival benefits and determine optimal dosing [87].

Brooks et al. conducted a real-world, retrospective study comparing IV edaravone-treated (n = 318) and untreated ALS patients (n = 318) between 2017 and 2020, with follow-up through 2021. Edaravone therapy extended mean survival to 29.5 months versus 23.5 months in controls, reducing mortality risk by ~27%. This study provides real-world evidence supporting edaravone’s survival benefit, complementing previous trials that showed slowed functional decline. Limitations include potential selection bias and non-randomized design. Nevertheless, findings underscore edaravone’s utility as an adjunct to riluzole, reinforcing its role in life-extension strategies for ALS [88].

Zebrafish models were used to recapitulate sporadic ALS-like phenotypes through exposure to bisphenol A (BPA) and β-methylamino-L-alanine (BMAA), followed by evaluation of edaravone’s neuroprotective effects. Behavioral, immunofluorescence, and metabolic analyses showed that BPA induced locomotor deficits and neuronal damage consistent with ALS pathology. Edaravone significantly mitigated these impairments, whereas riluzole demonstrated limited efficacy. Although zebrafish offer advantages for high-throughput screening, species-specific differences and short study durations limit direct translational relevance. Nonetheless, these findings support the use of zebrafish as a preclinical model for ALS drug discovery and highlight edaravone’s neuroprotective potential [89].

Thakore et al. analyzed the cost-effectiveness of riluzole using the FT9 (Fine’Til) staging system and a Markov model over 5–10 years, incorporating societal and healthcare perspectives. Recurring costs (RCs), such as medications and home care, dominated long-term expenses, whereas one-time costs (TCs) (e.g., hospitalizations) had lesser impact. Riluzole demonstrated favorable cost-effectiveness at $59,756/QALY (societal) and $67,658/QALY (healthcare), below the $100,000 threshold. Results reinforce riluzole’s economic value as a standard ALS therapy. Incorporating FT9 staging enables more precise cost–benefit analyses and supports policy planning for chronic ALS care. Future models should integrate longer time horizons and combination therapies [107].

Schuster et al. [109] previously reported a non-significant trend toward prolonged survival in ALS patients treated with rasagiline, a monoamine oxidase-B inhibitor, suggesting possible disease-modifying potential. To clarify these findings, a follow-up study [106] aimed to identify subgroups of ALS patients responsive to rasagiline and to assess the influence of dopaminergic SNPs (MOAB and DRD2) on treatment outcomes. A total of 122 ALS patients were treated for 18 months and stratified into very slow and intermediate-to-fast progressors based on ALSFRS-R decline. Clinical outcomes (ALSFRS-R, SVC, and survival) and genotyping were analyzed. Although no statistically significant survival benefit was observed within 18 months, rasagiline may confer delayed benefits in slow-progressing ALS subgroups. Additionally, SNP variants did not significantly alter treatment outcomes, though patients carrying the DRD2cc genotype (rs2283265) may exhibit enhanced responsiveness. These findings highlight the need for longer-duration studies and genotype-stratified trials to fully assess rasagiline’s therapeutic potential [106].

A clinical study evaluated the effects of ezogabine (retigabine), a Kv7 potassium channel opener, on motor neuron hyperexcitability in ALS. Sixty-five patients were randomized to receive placebo, 600 mg/day, or 900 mg/day ezogabine over 10 weeks. Cortical inhibition, measured by short-interval intracortical inhibition (SICI) using transcranial magnetic stimulation (TMS), served as the primary endpoint, alongside secondary assessments of spinal excitability, nerve conduction, and clinical progression. Ezogabine produced dose-dependent reductions in both cortical and spinal excitability, indicating effective target engagement, although no significant clinical improvement was observed. One serious adverse event (obtundation) occurred at the highest dose. These findings support ezogabine’s role as a neurophysiological modulator and highlight TMS and TTNCS as valuable pharmacodynamic biomarkers in ALS studies [102].

#### 5.2.2. Neuroinflammation-Targeted Therapies

Neuroinflammation is another major contributor to ALS progression, with increasing evidence implicating the cGAS-STING-TBK1 pathway in mediating inflammatory responses and MN degeneration. Targeting this signaling axis has therefore emerged as a potential strategy for mitigating neuroinflammation and disease progression. In this context, a recent study investigated Semen Strychni Pulveratum (SSP) and its active alkaloid vomicine, traditional Chinese medicinal compounds historically used for neuromuscular disorders, to determine their effects on ALS pathophysiology [92]. My collaborators and I have developed optimized methods to differentiate human iPSCs into MNs, providing a physiologically relevant platform for evaluating potential therapeutic targets [110,111,112,113,114,115]. Using such in vivo and in vitro models, the study examined the neuroprotective and anti-neuroinflammatory properties of SSP and vomicine. In hSOD1-G93A transgenic mice, oral SSP administration for eight weeks improved motor function, reduced body weight loss, and attenuated muscle atrophy and MN degeneration. Behavioral outcomes were complemented by histological analyses (H&E, Nissl staining) and molecular assays (Western blotting, RT-qPCR, immunofluorescence), which revealed suppression of cGAS-STING-TBK1 pathway activation and decreased expression of proinflammatory cytokines (IL-6, IL-1β). In hSOD1G93A NSC-34 MNs, vomicine treatment further confirmed the downregulation of cGAS-STING-TBK1 signaling. Molecular docking supported direct interactions between vomicine and key pathway proteins. Collectively, these results suggest that SSP and vomicine exert anti-neuroinflammatory and neuroprotective effects by modulating the cGAS-STING-TBK1 axis, thereby representing potential therapeutic candidates for ALS [92].

Another clinical investigation assessed the phosphodiesterase (PDE) inhibitor ibudilast (MN-166) for its ability to reduce glial activation and neuroaxonal loss in ALS, using PBR28-PET imaging and serum NfL as biomarkers [93]. The study rationale was based on prior evidence that PBR28-PET standardized uptake values (SUVR) and NfL levels remain stable without intervention but may decline in response to neuroprotective therapy. This open-label, phase 1b trial enrolled 35 ALS patients, who received ibudilast up to 100 mg/day for 36 weeks. Neuroinflammation was assessed using PBR28-PET (Siemens 3T Magnetom Tim Trio scanner), and serum NfL levels were quantified via Quanterix Simoa SR-X. Safety endpoints included adverse events and serious adverse events (SAEs), while exploratory outcomes included ALSFRS-R progression rates. Statistical analyses utilized Wilcoxon signed-rank and mixed-effects models. Despite acceptable tolerability, with 86% reporting adverse events and 31% discontinuation due to adverse events, no significant reductions in PBR28-PET SUVR or NfL levels were observed. These results indicate limited efficacy at the tested dose and duration. The study underscores the need for placebo-controlled, biomarker-driven trials, incorporating arterial sampling and adaptive platform designs to more effectively evaluate neuroinflammatory-targeted therapies in ALS [93].

Paganoni et al. [116] conducted a randomized, double-blind, placebo-controlled trial to evaluate sodium phenylbutyrate-taurursodiol, a fixed-dose co-formulation previously shown to reduce neuronal death in preclinical neurodegeneration models [117]. A total of 137 ALS patients were randomized 2:1 to treatment (n = 89) or placebo (n = 48) for 24 weeks. The primary outcome (ALSFRS-R slope) and secondary outcomes (Accurate Test of Limb Isometric Strength (ATLIS), SVC) were assessed alongside safety monitoring. Treatment was associated with a slower functional decline versus placebo, though differences did not reach statistical significance. GI disturbances were the most common adverse events. While these findings did not confirm definitive efficacy, they suggest potential clinical benefit warranting larger, longer trials [116].

Logan et al. [94] investigated ILB^®®^, a modified low molecular weight dextran sulfate (LMW-DS) that stimulates hepatocyte growth factor (HGF) release [118,119], in 13 ALS patients. Weekly subcutaneous ILB^®®^ injections (1 mg/kg) for 5 weeks improved vitality, mobility, and spasticity, with benefits persisting 3–4 weeks post-treatment. ILB^®®^ was safe, well tolerated, and reduced muscle atrophy biomarkers, indicating disease-modifying potential. These promising results warrant dose optimization and long-term evaluation [94].

In a post hoc analysis of the Phase 2b/3 AB10015 trial, masitinib (4.5 mg/kg/day) significantly slowed functional decline and extended both progression-free and overall survival by 9 and 12 months, respectively, in patients who had not yet experienced complete loss of any ALSFRS-R functionality. As a selective oral tyrosine kinase inhibitor, masitinib targeted the activation of mast cells and microglia, thereby modulating the neuroinflammatory microenvironment and reducing the pro-inflammatory signaling that drives MN degradation. These findings demonstrated that inhibiting these innate immune pathways in the early stages of ALS yielded a more favorable benefit-risk profile and successfully informed the patient stratification strategy for the confirmatory AB23005 study [120].

#### 5.2.3. Hormonal & Metabolic Modulation

Another avenue of investigation involves hormonal modulation, particularly the neuroprotective role of estrogen, given epidemiological evidence that premenopausal women exhibit a lower incidence of ALS and FTD than men or postmenopausal women. A recent study evaluated solid lipid curcumin particles (SLCP) as a non-hormonal estrogen replacement therapy to mitigate TDP-43-associated neuropathy in ALS [74]. Using Prp-TDP-43A315T transgenic mice, the study investigated the effects of SLCP on estrogen biosynthesis and disease progression. Western blotting, immunofluorescence, and confocal microscopy revealed that SLCP treatment enhanced estrogen levels by upregulating CYP19A1 (biosynthesis enzyme) and downregulating CYP3A4 (degradation enzyme), thereby improving estradiol homeostasis. SLCP also reduced the accumulation of pathological phosphorylated TDP-43 aggregates, improved survival, and mitigated body weight loss in female mice, though efficacy in male mice was limited, likely due to lower baseline estrogen levels. These findings suggest that SLCP may serve as an effective alternative to estrogen replacement therapy and support the role of CYP3A4 modulation in maintaining estrogen-mediated neuroprotection in ALS [74].

A muscle-to-spinal cord axis analysis revealed that FGF21 was markedly upregulated in atrophic myofibers and spinal tissues of ALS patients, with elevated plasma levels significantly correlating with slower disease progression and prolonged survival. These findings established FGF21 as a novel biomarker for clinical heterogeneity, as the protein demonstrated potent trophic effects by mitigating loss of cell viability in MNs and muscle cells under oxidative and proteotoxic stress. Mechanistically, FGF21 functioned as a metabolic hormone and neurotrophic factor that regulated glucose and lipid homeostasis while providing cytoprotection through the activation of its co-receptor, β-Klotho. The concomitant dysregulation of this FGF21 β-Klotho signaling pathway in both human samples and SOD1^G93A^ mice suggested that this axis played a critical role in the systemic response to neurodegeneration [121]. Delaye et al. examined whether fibroblast growth factor 21 (FGF21) pathway activation could mitigate ALS progression by reducing neuroinflammation and improving metabolic function. The FGF21 receptor agonist R1Mab1 was administered to SOD1^G93A^ mice at 12 and 16 weeks of age. Behavioral and metabolic assessments, including rotarod testing, cytokine profiling, and body weight monitoring, were conducted. Treated mice exhibited modest improvements in motor coordination (*p* = 0.032) and significant reductions in inflammatory markers. However, body weight loss (*p* = 0.001) indicated potential metabolic side effects. Overall, FGF21 pathway stimulation improved inflammatory and metabolic profiles, suggesting therapeutic potential in ALS [95].

#### 5.2.4. Protein Homeostasis & Aggregate Clearance

Given that TDP-43 phosphorylation and cytoplasmic mislocalization are hallmark features of ALS pathology, another study investigated whether inhibiting GSK-3β, a key kinase responsible for aberrant TDP-43 phosphorylation, could restore protein homeostasis and attenuate disease progression [75]. The GSK-3β inhibitor Tideglusib, previously tested in clinical trials for other neurological disorders, was evaluated in sporadic ALS lymphoblasts, SH-SY5Y neuroblastoma cells, and Prp-hTDP-43A315T transgenic mice. Immunoblotting and immunofluorescence analyses demonstrated that Tideglusib effectively reduced TDP-43 hyperphosphorylation, restored its nuclear localization, and prevented cytoplasmic aggregation. In ethacrynic acid-treated SH-SY5Y cells, Tideglusib also protected against TDP-43-induced cytotoxicity. In vivo, oral administration reduced phosphorylated TDP-43 levels in the spinal cord, confirming CNS penetration and pharmacological efficacy. These results support Tideglusib as a repurposable therapeutic candidate for ALS [75].

Another promising line of ALS research involves the development and characterization of a cellular model of endogenous TDP-43 sequestration, achieved by expressing a non-functional mutant TDP-43 that aggregates and sequesters the native protein. This model was employed for high-performance phenotypic screening to identify small molecules capable of enhancing TDP-43 aggregate clearance, a crucial therapeutic target in ALS-related proteinopathies. To evaluate translational potential, this approach was extended to a transgenic Drosophila model, enabling in vivo validation of candidate compounds. The primary objective was to demonstrate that thioridazine, a tricyclic antipsychotic compound, effectively promotes TDP-43 aggregate clearance through an autophagy-independent, proteasome-dependent mechanism, thereby restoring splicing functionality in vitro and improving locomotor performance in vivo. The cellular model expressing mutant TDP-43 facilitated fluorescence-based high-content screening, quantifying aggregate load after compound treatment. Aggregate clearance mechanisms were analyzed using biochemical fractionation and immunoblotting, while proteasome and autophagy inhibitors were applied to delineate the degradation pathway. Thioridazine treatment significantly enhanced TDP-43 aggregate clearance, restoring both in vitro splicing activity and in vivo motor function in Drosophila ALS models. Notably, tricyclic compounds, including thioridazine, reduced aggregation and rescued TDP-43-mediated functional deficits. The findings challenge the prior assumption that aggregate degradation is primarily autophagy-dependent, establishing a new paradigm for proteasome-mediated clearance. The Drosophila model further confirmed improved locomotor outcomes, underscoring thioridazine’s therapeutic potential in ALS. The study advocates for expanded high-performance screening to identify additional compounds with enhanced potency and selectivity [78].

Chen et al. [96] tested tamoxifen (40 mg/day), an autophagy enhancer, as an add-on to riluzole in 18 ALS patients (10 treatment, 8 placebo) without SOD1 or FUS mutations. Preclinical studies [122,123] indicated that tamoxifen reduces TDP-43 aggregation via autophagy activation. Over 12 months, the primary endpoint (survival or ventilation dependence) favored tamoxifen but was not statistically significant. ALSFRS-R decline was slower in the first 6 months, but group differences dissipated by month 12. Tamoxifen was safe and well tolerated, showing modest functional effects, highlighting the need for larger-scale trials [96].

#### 5.2.5. Gene-Targeted Therapies

WVE-004, a stereopure antisense oligonucleotide (ASO) is designed to selectively suppress pathogenic C9orf72 transcripts harboring G4C2 repeat expansions, while preserving physiological C9orf72 expression [77]. These pathogenic transcripts produce toxic RNA foci and dipeptide repeat proteins that drive neurodegeneration. The study aimed to evaluate variant-selective knockdown, target specificity, duration of action, and CNS distribution of WVE-004 in preclinical ALS models. In iPSC-derived MNs from C9orf72-ALS patients, WVE-004 treatment produced dose-dependent reduction of pathogenic transcripts as measured by qPCR. In C9 BAC transgenic mice, intracerebroventricular administration of WVE-004 at multiple doses significantly lowered poly(GP) biomarker levels and sustained transcript knockdown for at least six months post-injection. Importantly, C9orf72 protein levels remained stable, mitigating concerns about haploinsufficiency. RNA in situ hybridization confirmed widespread CNS distribution, while cytokine assays indicated minimal inflammatory responses. Pharmacokinetic analyses revealed a CNS half-life exceeding two months, supporting durable effects. Collectively, the data demonstrated that WVE-004’s variant-selective mechanism effectively suppresses toxic repeat-containing transcripts, positioning it as a promising therapeutic candidate for C9orf72-associated ALS and FTD [77].

Wei Cheng et al. investigated the therapeutic potential of AAV-mediated Neuron-Derived Neurotrophic Factor (NDNF) delivery in SOD1^G93A^ ALS model mice. The study aimed to determine whether targeted NDNF expression in the spinal cord could ameliorate motor deficits, prolong survival, and attenuate neurodegeneration. Using AAV-PHP.eB, an engineered capsid variant, NDNF was delivered via intrathecal (IT) injection to ensure expression within the spinal cord and brain. Advanced techniques, including CatWalk gait analysis, Western blotting, ELISA, and RNA sequencing, were employed. Early-stage treatment significantly improved motor function, gait smoothness, and lifespan, while preserving neuromuscular junctions and MN integrity. These findings highlight AAV-mediated NDNF therapy as a promising approach for ALS, offering neuroprotection and functional recovery. However, translational limitations remain due to interspecies differences, particularly reduced efficacy in cortical neurons, and the reliance on murine models. Further clinical studies are warranted to evaluate long-term safety, efficacy, and optimized delivery strategies in humans [124].

#### 5.2.6. Stem Cell & Cell-Based Therapies

Another therapeutic avenue involves cell-based therapy. Tavakol-Afshari et al. conducted a single-center, open-label, prospective trial to evaluate the safety and efficacy of autologous bone marrow-derived mesenchymal stem cells (BM-MSCs) delivered both IT and intravenously (IV) in ALS patients [97]. Fifteen participants (aged 18–75 years) meeting El Escorial diagnostic criteria underwent BM aspiration from the iliac crest, with cells expanded under GMP conditions and verified for sterility. Each patient received 1 × 10^6^ cells/kg IT (L3–L4) and IV infusion of the same dose. Patients were followed for six months, with ALSFRS-R and forced vital capacity (FVC) as primary outcomes. Mild headache, allergic reactions, and transient urinary symptoms were reported, but no SAEs occurred. Functional measures remained stable for three months, followed by decline at six months, suggesting transient benefits. The findings indicate that combined IT and IV BM-MSC administration is safe and may offer short-term disease stabilization, supporting repeated dosing strategies and larger controlled trials to validate long-term efficacy [97]. Ciervo et al. evaluated adipose-derived stem cells (ADSCs) as a potential therapy for ALS, focusing on their paracrine neuroprotective mechanisms. In vitro, ADSCs shielded MNs from astrocyte-mediated toxicity via secretion of VEGF and IGF-1, while in vivo administration in SOD1^G93A^ mice delayed disease onset, preserved motor function, and reduced neuroinflammation. Rather than neuronal replacement, ADSCs exerted effects through trophic support and immune modulation. Although animal data are encouraging, translation to humans remains challenging due to heterogeneity of ALS pathology and limited understanding of long-term outcomes. Larger, controlled trials are required to establish safety, dosing regimens, and mechanistic durability [98]. Moreover, the safety and feasibility of transplanting engineered human neural progenitor cells (CNS10-NPC-GDNF) that produce GDNF into the lumbar spinal cords of patients with ALS is investigated. This first-in-human study aimed to determine whether localized GDNF delivery could slow disease progression by providing long-term trophic support. Eighteen ALS patients were randomized into low-dose (n = 9) and high-dose (n = 9) cohorts and underwent unilateral transplantation into the spinal cord intermediate zone to avoid direct MN injury. One leg served as the treated side, while the contralateral leg served as an internal control. Over a 42-month follow-up, the primary endpoint (safety) was assessed via adverse event monitoring and ALS Functional Rating Scale-Revised (ALSFRS-R) scores, while secondary endpoints (efficacy) included graft survival and GDNF expression determined through histopathology and immunohistochemistry. Transplanted cells survived for up to 42 months, continued to produce GDNF, and did not cause adverse motor effects. These findings demonstrated the long-term viability and safety of CNS10-NPC-GDNF grafts, supporting this strategy as a promising cell-based therapeutic for ALS and other neurodegenerative diseases [99].

In addition, Cudkowicz et al. [100] assessed autologous mesenchymal stem cells induced to secrete neurotrophic factors (MSC-NTF) in 189 ALS patients (MSC-NTF, n = 95; placebo, n = 94) receiving repeated IT injections over 16 weeks. The primary endpoint (ALSFRS-R slope) was unmet, but treatment was well tolerated, with biomarker analysis confirming neurotrophic and anti-inflammatory effects (increase in VEGF and decrease in MCP-1 and NfL). MSC-NTF represents a biologically active, safe intervention, though efficacy remains unproven [125]. Also, Petrou et al. [101] earlier demonstrated the safety and tolerability of single intramuscular (IM) or IT MSC-NTF administration in phase 1/2 and 2a studies involving early- and late-stage ALS patients (n = 26). While no significant efficacy was established, IT and IM routes showed functional trends, supporting further dose-optimization trials [101].

#### 5.2.7. Receptor Modulation & Novel Signaling Targets

EphA4, a receptor tyrosine kinase, has emerged as a genetic and pharmacological disease modifier in ALS. Elevated EphA4 expression accelerates disease progression, whereas loss-of-function variants confer a survival advantage. However, prior strategies focusing on EphA4 inhibition failed to yield significant benefit, potentially because unbound EphA4 can trigger pro-apoptotic signaling. To overcome this limitation, a recent study investigated synthetic EphA4 agonists (123C4, 150D4, 150E7, 150E8) that stabilize ligand-bound conformations, thereby attenuating astrocyte-mediated MN toxicity [103]. Using NMR spectroscopy and isothermal titration calorimetry (ITC), the agonists were confirmed to bind the EphA4 ligand-binding domain with high affinity. Patient-derived NPC astrocyte-MN co-culture systems were used to evaluate neurotoxicity, and HTS (384-well format) identified the most potent compounds. Western blotting and immunoprecipitation assessed EphA4 phosphorylation, while MN survival assays quantified efficacy. The results revealed that second-generation EphA4 agonists provided dose-dependent neuroprotection, reversing astrocyte-induced MN death. The findings confirm that EphA4 activation, rather than inhibition, may represent a novel therapeutic approach, with 150E7 and 150E8 emerging as strong candidates for further preclinical development [103].

Imamura et al. conducted a phase I clinical trial assessing Bosutinib safety, tolerability, and biomarker response in 20 ALS patients across four Japanese centers. Doses up to 300 mg/day were well-tolerated, while 400 mg/day induced dose-limiting toxicities (hepatotoxicity, rash). Notably, patients with lower baseline NFL levels exhibited superior clinical responses, suggesting NFL as a predictive biomarker for treatment stratification. Despite the small cohort and short duration (20 weeks), findings support further dose-optimized, biomarker-guided phase II/III studies to determine efficacy and long-term safety [104].

The long-term safety and tolerability of oral edaravone, developed as a convenient alternative to IV administration, is evaluated [90]. Previous pharmacological [126] and bioequivalence studies [127] established that 105 mg oral edaravone achieves exposure equivalent to 60 mg IV dosing, which is approved for ALS. In a 48-week open-label trial, 185 ALS patients received cyclic 4-week courses of oral edaravone. Safety endpoints (treatment-emergent adverse events (TEAEs), SAEs, laboratory tests, ECGs, and Columbia-Suicide Severity Rating Scale (C-SSRS)) and functional measures (ALSFRS-R, FVC) were evaluated at 24 and 48 weeks. Oral edaravone was well tolerated, with no new safety concerns compared to IV formulation. Common side effects included fatigue, dizziness, headache, and constipation. These results support oral edaravone as a safe, effective, and more accessible alternative for ALS management [90]. Furthermore, Shefner et al. [105] evaluated reldesemtiv, a fast skeletal muscle troponin activator (FSTA), in a 12-week, phase 2 trial involving 457 ALS patients randomized to 150, 300, or 450 mg BID or placebo. Primary (SVC) and secondary (ALSFRS-R, muscle strength megascore) endpoints were not met (*p* > 0.05), though consistent trends toward benefit were observed across all efficacy measures. Reldesemtiv was well tolerated, with nausea and fatigue being the most frequent adverse events. These results suggest biological activity but inconclusive efficacy, warranting further evaluation in extended studies [105,128,129]. However, recently, the Phase 3 COURAGE-ALS randomized clinical trial failed to demonstrate the efficacy of reldesemtiv in slowing functional decline, leading to the early termination of the study for futility after a 24-week interim analysis. Comparison between the treatment and placebo groups revealed no significant improvement in ALSFRS-R scores or combined assessments of function and survival, with data unexpectedly favoring the placebo arm in some functional measures (*p* = 0.04). As a FSTA, reldesemtiv was designed to slow the release of calcium from the troponin complex in fast-twitch muscle fibers, thereby sensitizing the sarcomere to calcium and increasing muscle contractility in response to diminished MN signaling. Despite the strong mechanistic rationale of augmenting peripheral muscle strength to compensate for neuromuscular denervation, these clinical findings indicated that such physiological enhancement did not translate into a durable therapeutic benefit for patients with ALS [130].

#### 5.2.8. Neurophysiological and Functional Biomarkers

Govaarts et al. examined resting-state brain activity via magnetoencephalography (MEG) in early-stage ALS (n = 34), bvFTD (n = 18), and healthy controls (n = 18) to investigate shared neurophysiological signatures. ALS and bvFTD patients displayed increased delta (slow) and gamma (fast) band activity in frontal, limbic, and subcortical regions, suggesting overlapping network dysfunctions. These findings support the ALS-FTD disease spectrum hypothesis, linking motor, cognitive, and emotional impairments to common cortical-subcortical disruptions. Although longitudinal data are lacking, MEG biomarkers hold promise for early diagnosis and differentiation of ALS phenotypes [108].

### 5.3. Critical Perspective Across New Therapeutic Strategies in ALS

Overall, the oxidative stress and neuroprotective modulation landscape in ALS reflects a highly active but translationally constrained field in which strong mechanistic rationale repeatedly outpaces clinical validation. Multitarget fasudil derivatives combining ROCK inhibition with NRF2 activation represent an elegant rational design strategy, yet their evidence base remains largely confined to reductionist in vitro systems, including KEAP1-modulated reporter assays and lymphoblastoid cell models, which may overestimate NRF2 pathway engagement and do not adequately capture the multicellular and non–cell-autonomous nature of ALS pathology; moreover, the observed selective activity in SOD1-ALS–derived cells but not sporadic ALS models raises concerns about model dependency rather than true genotype-stratified efficacy. Similarly, fasudil itself demonstrates a clear safety profile in a rigorous phase 2 randomized controlled trial, but its extremely short treatment window and modest exploratory biomarker signals (e.g., MUNIX trends and sex-specific SVC effects) limit any meaningful inference of disease modification, instead reinforcing the notion that ROCK inhibition alone is insufficient without optimized dosing, chronic exposure, or validated combination strategies. The AI-identified nebivolol–donepezil combination further illustrates the gap between mechanistic plausibility and ALS relevance, as modulation of NF-κB and PI3K-Akt signaling in SOD1G93A mice primarily reflects broad anti-inflammatory and pro-survival signaling rather than ALS-specific disease mechanisms, and its reliance on familial ALS models substantially limits generalizability to sporadic disease. Real-world and registry-based signals from TUDCA and edaravone, while clinically encouraging, are heavily confounded by retrospective design, selection bias, and survivorship effects, with survival extensions that may be inflated relative to controlled trial contexts and therefore best interpreted as hypothesis-generating rather than confirmatory evidence. Preclinical zebrafish models demonstrating edaravone neuroprotection further support antioxidant effects but are constrained by simplified neurobiology and toxin-induced phenotypes that do not replicate human ALS pathogenesis, limiting translational fidelity. Rasagiline and ezogabine studies highlight a recurring disconnect between biomarker modulation (e.g., SICI reduction, excitability changes) and clinical outcomes, underscoring that electrophysiological or molecular target engagement does not necessarily translate into functional neuroprotection, particularly when subgroup findings and SNP associations remain underpowered and vulnerable to false-positive interpretation. Collectively, these studies converge on a central limitation of the oxidative stress–targeted therapeutic paradigm in ALS: while oxidative and inflammatory pathways are clearly involved in disease progression, their modulation in isolation yields inconsistent or modest effects, likely due to disease heterogeneity, late-stage intervention, and incomplete targeting of upstream pathogenic drivers such as TDP-43 proteinopathy, RNA dysregulation, and non-neuronal cell contributions, ultimately emphasizing that NRF2/ROCK-centered strategies may function better as adjunctive rather than standalone disease-modifying approaches.

### 5.4. Last Point

Recent research in ALS has explored a broad range of therapeutic strategies, including gene therapy, pharmacological interventions, trophic factor–based approaches, and stem cell–derived treatments. Gene therapy has demonstrated meaningful improvements in motor function and survival in preclinical models, particularly through targeting pathogenic mechanisms such as C9orf72-related toxicity, but its efficacy in cortical and broader CNS neuronal populations remains variable, and further optimization of delivery and long-term expression is required. Similarly, trophic factor–based strategies such as FGF21 signaling modulation have shown anti-inflammatory and metabolic benefits, yet concerns such as unintended weight loss highlight challenges in balancing efficacy with systemic effects. Drug-based approaches, including combinations and repurposed agents like TUDCA and IV edaravone, have demonstrated modest survival benefits and functional stabilization in some cohorts; however, their long-term efficacy, durability, and safety profiles remain incompletely defined. Stem cell therapies have shown promise in supporting motor neuron survival and modulating the inflammatory microenvironment, but their clinical translation is limited by heterogeneity in patient response, delivery challenges, and transient therapeutic effects.

Overall, while many of these interventions demonstrate biological plausibility and encouraging early signals, the most consistently promising therapeutic directions appear to be those targeting upstream and convergent disease mechanisms rather than isolated symptomatic pathways. Strategies focused on core pathogenic nodes, such as TDP-43 proteinopathy and proteostasis (like GSK-3β inhibition and aggregate clearance approaches), neuroinflammatory signaling (including cGAS–STING–TBK1 and mast cell/microglial modulation such as masitinib), and genetic drivers (such as C9orf72-selective antisense oligonucleotides), show stronger translational coherence due to converging mechanistic, preclinical, and early clinical support. In contrast, broadly neuroprotective or antioxidant-based therapies, although generally safe and mechanistically relevant, tend to yield inconsistent clinical outcomes, likely reflecting disease heterogeneity and late-stage intervention effects. Collectively, the field increasingly supports a shift toward combination and precision-based therapeutic frameworks that integrate gene-targeted, anti-inflammatory, and cell-based strategies while incorporating adjunctive neuroprotective agents, alongside the necessity for long-term, well-controlled clinical trials to validate safety, durability, and efficacy across distinct ALS subtypes.

## 6. Conclusions and Future Directions

**Figure 3:**
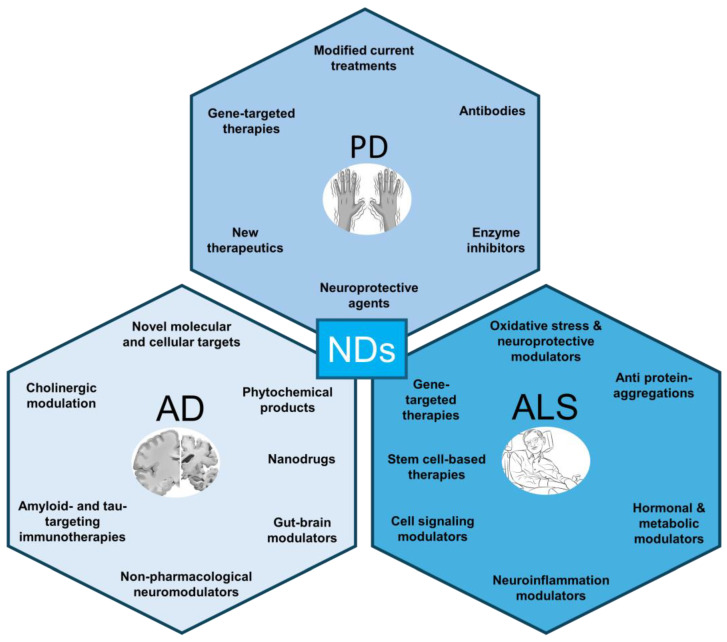
Integrated therapeutic strategies for major neurodegenerative diseases.

To integrate the diverse approaches discussed in this Review, we provide a schematic overview (Figure 3) that organizes the major therapeutic strategies across these three neurodegenerative diseases into a unified framework. The management of Alzheimer’s disease, Parkinson’s disease, and amyotrophic lateral sclerosis requires an integrative and multidisciplinary approach that combines pharmacological, non-pharmacological, and emerging therapeutic strategies. Despite advances in clinical trial design, biomarker development, and preclinical modeling, significant challenges remain in translating experimental findings into meaningful clinical outcomes. Heterogeneity in disease progression, variability in trial endpoints, and limited demographic representation further complicate the development of effective therapies. Current treatments provide symptomatic relief but often fail to achieve sustained disease modification. Emerging approaches, including gene-targeted therapies, neuromodulation, nanodelivery systems, and precision medicine, offer promising avenues for more individualized and effective interventions. However, issues such as long-term efficacy, safety, and accessibility must be addressed.

Future research should prioritize optimizing treatment timing, standardizing outcome measures, enhancing multimodal assessments, and improving drug delivery systems. A greater emphasis on personalized, biomarker-driven strategies and inclusive clinical trial design will be essential. Collectively, these efforts hold the potential to slow disease progression, preserve neurological function, and improve the quality of life for patients affected by these devastating neurodegenerative disorders.

## Figures and Tables

**Table 1:** Emerging therapeutic strategies for Alzheimer’s disease (AD).

Authors	Type of Study	Treatment	Mechanism	Model	Efficacy	Safety	Key Findings
***Cholinergic modulation and symptomatic therapies***							
Hussain et al. [8]	Experimental	Benzoxazole-based oxazole derivatives	AChE and BuChE inhibition	In vitro enzyme assays; molecular docking	IC50: 0.90 µM (AChE), 1.10 µM (BuChE)	Not reported	Novel cholinesterase inhibitors with high potency
Valis et al. [24]	Clinical pharmacokinetics	Donepezil (10 mg/day)	AChE inhibition; CNS accumulation	40 AD patients	Increased CNS exposure over time	Well tolerated	CSF/plasma ratio may guide dosing
Farlow et al. [22]	Phase 3 RCT	Donepezil (23 mg vs. 10 mg)	Dose-dependent AChE inhibition	1371 AD patients	Improved cognition in severe AD	GI side effects increased	Higher dose improves efficacy but increases AEs
***Amyloid- and tau-targeting immunotherapies***							
Sperling et al. [17]	Phase 3 RCT	Solanezumab	Anti-Aβ antibody	1169 early AD patients	No significant benefit	Safe	Failed to slow progression
Van Dyck et al. [25]	Phase 3 RCT	Lecanemab	Anti-Aβ fibrils	1795 early AD patients	Mixed clinical benefit	ARIA risk	Reduces amyloid burden
Florian et al. [18]	Phase 2 RCT	Tilavonemab	Anti-tau antibody	453 AD patients	No clinical efficacy	Safe	Target engagement only
Rojas [26]	Phase 3 extension	Donanemab	Amyloid plaque removal	Early AD patients	Slower decline in early stage	ARIA risk	Stage-dependent efficacy
***Novel molecular and cellular targets***							
Musardo et al. [19]	Preclinical	PEP3 peptide	ADAM10 modulation	APP/PSEN1 mice	Improved synaptic function	Not reported	Early intervention effective
Nada et al. [27]	Preclinical	S9 agonist	TREM2 activation	iPSC microglia	Reduced inflammation	Improved PK	Oral TREM2 agonist
Dubois et al. [16]	Phase 3 RCT	Masitinib	Tyrosine kinase inhibition	718 AD patients	Reduced decline	Dose-related AEs	First positive kinase trial
Yang et al. [28]	Preclinical	AAV APOE therapy	APOE modulation	AD mice	Improved cognition	Not reported	Timing-dependent efficacy
***Nanotechnology-based therapies***							
El-Hawwary et al. [29]	Experimental	ZnO nanoparticles	AChE inhibition	In vitro + animals	Comparable to donepezil	Biocompatible	Green nanotherapy
Yan et al. [30]	Preclinical	Microneedle patch	Sustained delivery	AD models	Improved cognition	Safer PK	Enhanced bioavailability
Liu et al. [31]	Preclinical	CO-MOF nanozyme	Anti-inflammatory	AD mice	Improved memory	Low toxicity	Neuroinflammation targeting
Zhou et al. [21]	Preclinical	Hydrogel delivery	Controlled release	Mouse AD model	Improved memory	Safe	Reduced GI side effects
***Natural products and phytochemicals***							
Wang et al. [32]	In silico	*D. discoideum* metabolites	Multi-target anti-inflammatory	Network pharmacology	Strong binding	Not tested	COX-2 and TNF-α targeting
***Gut–brain axis modulation***							
Den et al. [12]	Meta-analysis	Probiotics	Microbiome modulation	297 participants	Improved cognition (SMD 0.37)	Safe	Reduced inflammation
***Non-pharmacological interventions***							
Moussavi et al. [23]	Phase 3 RCT	rTMS	Synaptic plasticity	135 AD patients	Short-term benefit only	Safe	No long-term effect
Yu et al. [11]	RCT	Aerobic exercise	Neuroplasticity	96 AD patients	Trend benefit	Safe	No significant difference
Gómez-Gallego et al. [9]	Quasi-experimental	Music therapy	Cognitive stimulation	90 patients	AMI improved cognition	Safe	Motor and cognitive benefit
Koch et al. [15].	Phase 2 RCT	Precuneus rTMS	Network modulation	50 AD patients	Improved MMSE	Mild AEs	Promising targeted rTMS

**AAV**, adeno-associated virus; **Aβ**, amyloid-beta; **AD**, Alzheimer’s disease; **ADAM10**, a disintegrin and metalloproteinase 10; **AChE**, acetylcholinesterase; **APOE**, apolipoprotein E; **APP/PSEN1**, amyloid precursor protein/presenilin 1; **AEs**, adverse events; **BuChE**, butyrylcholinesterase; **CNS**, central nervous system; **CO-MOF**, cobalt–metal organic framework; **COX-2**, cyclooxygenase-2; **CSF**, cerebrospinal fluid; **GI**, gastrointestinal; **iPSC**, induced pluripotent stem cell; **MMSE**, Mini-Mental State Examination; **PK**, pharmacokinetics; **RCT**, randomized controlled trial; **rTMS**, repetitive transcranial magnetic stimulation; **SMD**, standardized mean difference; **TNF-α**, tumor necrosis factor alpha; **TREM2**, triggering receptor expressed on myeloid cells 2; **ZnO**, zinc oxide.

**Table 2:** Emerging and novel therapeutic strategies in Parkinson’s Disease (PD).

Authors	Type of Study	Treatment	Mechanism	Model	Efficacy	Safety	Key Findings
***Modified current treatments***							
Rosebraugh et al. [48]	Preclinical + clinical	Foslevodopa/foscarbidopa (s.c. infusion)	Continuous dopaminergic stimulation	Animals + PD patients	Stable plasma LD; reduced motor fluctuations	Feasible; device-dependent	Improved pharmacokinetics vs. oral LD/CD
Olanow et al. [49]	Clinical	ND0612 (s.c. LD/CD infusion)	Continuous dopamine delivery	PD patients	↓ off-time (~2 h); ↑ on-time (~3.3 h)	Mild AEs; local reactions	Consistent symptom control; improved sleep
Liu et al. [50]	Experimental	Nardosinone + Levodopa	Gut–brain modulation; dopamine synthesis	Rotenone PD rats	Improved motor + neuronal protection	Not reported	Synergistic effect via microbiota
***Antibodies***							
Pagano et al. [51]	Phase 2	Prasinezumab	Anti-α-synuclein antibody	Early PD patients	Slower progression trends	Well tolerated	Potential disease-modifying effect
***Enzyme inhibitors***							
Peterschmitt et al. [52]	Clinical	Venglustat	Glucosylceramide synthase inhibition	GBA-PD patients	↓ GL-1 levels (dose-dependent)	Acceptable safety	BBB penetration; target engagement
Neumann et al. [53]	Phase 3	Rivastigmine (patch)	AChE inhibition	PD patients	Ongoing	Ongoing	Evaluates fall reduction and cognition
Minnella et al. [54]	Experimental	PTC-041	15-LO inhibition; anti-ferroptosis	Rodent PD models	Improved motor + neuronal survival	Not reported	Reduces α-syn aggregation
***Neuroprotective agents***							
Li et al. [55]	Experimental	(-)-Clausenamide	Anti-ferroptosis; antioxidant	MPTP mice + neurons	Improved motor; ↓ lipid peroxidation	Not reported	Targets PKCα-ALOX5 pathway
Kim et al. [56]	Experimental	Fexofenadine	Anti-inflammatory; microglial modulation	PD mouse models	↓ α-syn; improved motor	Safe	Repurposable antihistamine
Zhou et al. [57]	Experimental	Ceftriaxone	Gut–brain axis; neurotrophic support	MPTP mice	Improved motor; ↓ inflammation	Not reported	Restores microbiota and BDNF/GDNF
Wu et al. [58]	Experimental	Ginkgo biloba + PCA	Antioxidant synergy	Cell + MPTP mice	↑ neurons; ↓ ROS	Not reported	Synergistic neuroprotection
Takeshige-Amano et al. [59]	Clinical	Selegiline	MAO-B inhibition; WM protection	PD patients	Reduced WM abnormalities	Well tolerated	Preserves brain microstructure
Shang et al. [60]	Experimental	FLZ	Microbiota-dependent metabolism	MPTP mice	Improved neuroprotection	Not reported	Gut microbiota critical for efficacy
Knez et al. [61]	Experimental	Azastilbene derivatives	Selective MAO-B inhibition	Cell + mice	Improved motor; ↓ oxidative stress	Not reported	Next-gen MAO-B inhibitors
Cao et al. [62]	Experimental	Compound 3h	MAO-B inhibition + antioxidant	Cell models	Neuroprotection	Not reported	Multi-target BBB-penetrant
***New therapeutic strategies***							
Sun et al. [63]	Experimental	Ursolic acid	Anti-apoptotic + anti-inflammatory	MPTP mice + SH-SY5Y	Improved motor; ↓ cytokines	Limited bioavailability	Multi-target candidate
Dai et al. [64]	Experimental	Dendrimer-FN nanocomplex	Microglial modulation (M1→M2)	MPTP mice	Improved motor; ↓ α-syn	Not reported	BBB-targeted delivery
Alimohammadi et al. [65]	Computational	Graphene nanoparticles	Inhibit α-syn fibrillation	Molecular simulation	Reduced aggregation	Not reported	N-graphene most effective
LUMA study [66]	Phase 2	BIIB122 (DNL151)	LRRK2 inhibition	PD patients	Target engagement	Ongoing	Precision medicine approach
***Genomic evaluations***							
Liu et al. [67]	Experimental	DEDRG targets	Cell death pathway modulation	PD mice	Biomarker potential	Not reported	ACTB, ACTN4,, etc., identified
Stom et al. [68]	Genetic study	Drug-target genes	Genetic causal inference	GWAS datasets	Identified targets	Not applicable	CTSB, CD38 promising
Wang et al. [69]	Experimental	SNAP25 targeting	Synaptic dysfunction	AD/PD mice	Altered expression	Not reported	Shared AD–PD biomarker
Park et al. [70]	Experimental	PAANIB-1	Parthanatos inhibition	PD mice	Neuroprotection	Not reported	Targets MIF nuclease
Quan et al. [71]	Computational	Repurposed drugs	Network pharmacology	Databases	Identified candidates	Not applicable	EGFR/HDAC targets

**15-LO**, 15-lipoxygenase; **AChE**, acetylcholinesterase; **AD**, Alzheimer’s disease; **AEs**, adverse events; **ALOX5**, arachidonate 5-lipoxygenase; **BBB**, blood–brain barrier; **BDNF**, brain-derived neurotrophic factor; **CD**, carbidopa; **DEDRG**, differential expression disulfidptosis-related genes; **EGFR**, epidermal growth factor receptor; **FN**, fibronectin; **GBA**, glucocerebrosidase gene; **GDNF**, glial cell line-derived neurotrophic factor; **GL-1**, glucosylceramide; **GWAS**, genome-wide association studies; **HDAC**, histone deacetylase; **LD**, levodopa; **LD/CD**, levodopa/carbidopa; **LRRK2**, leucine-rich repeat kinase 2; **MAO-B**, monoamine oxidase B; **MIF**, macrophage migration inhibitory factor; **MPTP**, 1-methyl-4-phenyl-1,2,3,6-tetrahydropyridine; **PAAN**, parthanatos-associated apoptosis-inducing factor nuclease; **PCA**, protocatechuic acid; **PD**, Parkinson’s disease; **PKCα**, protein kinase C alpha; **ROS**, reactive oxygen species; **s.c.**, subcutaneous; **SNAP25**, synaptosomal-associated protein 25; **WM**, white matter; **α-syn**, α-synuclein.

**Table 3:** The novel treatments of ALS.

Authors	Type of Study	Treatment	Mechanism	Model	Efficacy	Safety	Key Findings
***Oxidative stress and antioxidant therapies***							
Martic-Camara et al., [76]	Experimental	Multitarget hybrid fasudil derivatives	ROCK inhibition + NRF2 activation via KEAP1	HEK293T, SH-SY5Y, MEF cells; SOD1-ALS lymphoblasts	Compound 1d increase NRF2-dependent genes; genotype-specific efficacy (SOD1 > sALS)	Not reported	Promising lead for SOD1-ALS; needs PK/ADMET optimization
Koch et al., [86]	Phase 2 RCT	Fasudil	ROCK inhibition	115 ALS pts (Germany, France, Switzerland)	No ALSFRS-R improvement; possible MUNIX signal; slower SVC decline in females	Well tolerated; no serious AEs	Safe; longer, higher dose/oral trials needed
Zucchi et al., [87]	Retrospective	TUDCA (oral)	Mitochondrial/anti-oxidative	86 treated, 172 controls	Increase Survival (56.5 vs. 36.2 mo), stable ALSFRS-R	Mild GI, skin effects	High-dose TUDCA safe; supports adjunctive use
Brooks et al., [88]	Retrospective	Edaravone (IV)	ROS scavenger	318 treated vs. 318 controls	Increase Survival (29.5 vs. 23.5 mo)	Safe	Supports edaravone as adjunct to riluzole
Oliveira et al., [89]	Experimental	Edaravone	Antioxidant	BPA/BMAA-induced zebrafish	Improved motor/neural functions	—	Validates zebrafish ALS model
Genge et al. [90]	48-week open-label	Edaravone (oral 105 mg)	Antioxidant	185 ALS pts	Stable ALSFRS-R, FVC	Mild fatigue, headache	Oral = IV efficacy; convenient
***Neuroinflammation modulators***							
Lee et al., [91]	Experimental	Nebivolol + Donepezil	NF-κB inhibition, PI3K-Akt activation	SOD1^G93A^ mice; BV-2 cells	Decrease cytokines, increase survival	—	Synergistic neuroprotection
Zhang et al., [92]	Experimental	SSP & Vomicine (Chinese medicine)	cGAS-STING-TBK1 inhibition	SOD1-G93A mice	Increase motor, decrease cytokines	—	Anti-inflammatory via STING modulation
Babu et al., [93]	Phase 1b	Ibudilast	PDE inhibition (glial suppression)	35 ALS pts	No significant decrease PBR28-PET SUVR/NfL	86% AEs; 31% discontinued	Limited efficacy; biomarker trial
Logan et al., [94]	Pilot	ILB^®^ (LMW dextran sulfate)	HGF pathway activation	ALS pts	Increase vitality, decrease atrophy markers	Safe	Short-term benefits
***Hormonal regulation and estrogenic pathways***							
Majumder et al., [74]	Experimental	SLCP (Curcumin formulation)	Increase Estrogen biosynthesis (increase CYP19A1, decrease CYP3A4)	TDP-43A315T mice	Increase E2, decrease pTDP-43, increase survival (female)	—	Female-specific neuroprotection
Delaye et al., [95]	Experimental	FGF21 agonist (R1Mab1)	Improved inflammatory and metabolic profiles	SOD1^G93A^ mice	Increase motor function, decrease cytokines	Weight loss	Dose balance critical
***Protein homeostasis & autophagy***							
Martinez-Gonzalez et al., [75]	Experimental	Tideglusib	GSK-3β inhibition → decrease TDP-43 phosphorylation	ALS lymphoblasts, SH-SY5Y, TDP-43 mice	Restored nuclear TDP-43	Safe (preclinical)	Repurposable GSK-3β inhibitor
Cragnaz et al., [78]	Experimental	Thioridazine	Proteasome-dependent TDP-43 clearance	Cell & Drosophila ALS models	Decrease aggregates, increase locomotion	—	Autophagy-independent, proteasome-mediated
Chen et al., [96]	Phase 2	Tamoxifen + Riluzole	Autophagy enhancer	18 ALS pts	Slower ALSFRS-R decline (6 mo)	Safe	Mild functional benefit
***Gene-targeted and RNA therapies***							
Liu et al., [77]	Preclinical	WVE-004 (ASO)	C9orf72 repeat knockdown (variant-selective)	C9 iPSC-MNs; BAC mice	Decrease toxic transcripts, decrease poly(GP), preserved C9 protein	Minimal inflammation	Sustained CNS effect; promising for C9-ALS
***Cell-based therapies***							
Tavakol-Afshari et al., [97]	Open label	BM-MSCs (IT + IV)	Paracrine support	15 ALS pts	Stable 3 mo; decline by 6 mo	Mild AEs	Safe; short-lived benefit
Ciervo et al., [98]	Preclinical	ADSCs	VEGF & IGF-1 secretion	SOD1^G93A^ mice	Decrease onset, increase motor	—	Paracrine trophic support
Baloh et al., [99]	Phase 1	CNS10-NPC-GDNF	GDNF-secreting NPC grafts	18 ALS pts	Long-term graft survival	No adverse motor effects	Safe, localized support up to 42 mo
Cudkowicz et al., [100] Petrou et al., [101]	Phase 2	MSC-NTF (NurOwn^®®^)	Neurotrophic + anti-inflammatory	189 ALS pts	ALSFRS-R slope unmet; increase VEGF, decrease MCP-1	Well tolerated	Biologically active, efficacy modest
***Receptor and channel modulation***							
Wainger et al., [102]	Phase 2	Ezogabine	Kv7 channel opener	65 ALS pts	Decrease excitability (dose-dependent)	1 SAE (obtundation)	Biomarker validation (TMS, TTNCS)
Dennys et al., [103]	Experimental	EphA4 agonists (150E7, 150E8)	EphA4 activation (ligand-bound stabilization)	Astrocyte-MN co-culture	Dose-dependent neuroprotection	—	Reverses astrocyte toxicity
Imamura et al., [104]	Phase 1	Bosutinib	Src/c-Abl inhibition	20 ALS pts (Japan)	Improved outcomes (low-NfL subgroup)	400 mg: hepatotoxicity, rash	Biomarker-guided dosing
Shefner et al., [105]	Phase 2	Reldesemtiv	Troponin activator (skeletal muscle)	457 ALS pts	No significant endpoints; benefit trends	Nausea, fatigue	Longer trials warranted
Schuster et al., [106]	Follow-up	Rasagiline	MAO-B inhibition	122 ALS pts	Trend in slow progressors	Well tolerated	Genotype (DRD2cc) responsive
***Adjunctive or supportive interventions***							
Oliveira et al., [107]	Economic analysis	Riluzole	Glutamate inhibition	Markov model (FT9 staging)	$59,756/QALY (societal)	Cost-effective	Reinforces riluzole’s value
***Neuroimaging and biomarker studies***							
Govaarts et al., [108]	Cross-sectional	MEG	Resting-state delta/gamma activity	ALS (n = 34), bvFTD (n = 18), controls (n = 18)	Increase delta/gamma activity	Safe	Supports ALS-FTD spectrum

**ADMET**, absorption, distribution, metabolism, excretion, and toxicity; **ADSCs**, adipose-derived stem cells; **AE**, adverse event; **ALS**, amyotrophic lateral sclerosis; **ALSFRS-R**, ALS functional rating scale-revised; **ASO**, antisense oligonucleotide; **BAC**, bacterial artificial chromosome; **BMAA**, β-N-methylamino-L-alanine; **BM-MSCs**, bone marrow-derived mesenchymal stem cells; **BPA**, bisphenol A; **BV-2**, murine microglial cell line; **C9 iPSC-MNs**, C9orf72 induced pluripotent stem cell-derived motor neurons; **C9orf72**, chromosome 9 open reading frame 72; **cGAS**, cyclic GMP-AMP synthase; **CNS**, central nervous system; **CYP19A1**, cytochrome P450 family 19 subfamily A member 1 (aromatase); **CYP3A4**, cytochrome P450 family 3 subfamily A member 4; **Drosophila**, fruit fly model organism; **DRD2**, dopamine receptor D2; **E2**, estradiol; **EphA4**, ephrin type-A receptor 4; **FGF21**, fibroblast growth factor 21; **FT9**, fine-tuned 9-stage model; **FTD**, frontotemporal dementia; **FVC**, forced vital capacity; **GDNF**, glial cell line-derived neurotrophic factor; **GI**, gastrointestinal; **GSK-3β**, glycogen synthase kinase 3 beta; **HEK293T**, human embryonic kidney 293T cells; **HGF**, hepatocyte growth factor; **IGF-1**, insulin-like growth factor 1; **iPSC**, induced pluripotent stem cell; **ILB^®®^**, low molecular weight dextran sulfate; **IT**, intrathecal; **IV**, intravenous; **KEAP1**, kelch-like ECH-associated protein 1; **LMW**, low molecular weight; **MAO-B**, monoamine oxidase B; **MCP-1**, monocyte chemoattractant protein-1; **MEF**, mouse embryonic fibroblast; **MEG**, magnetoencephalography; **mo**, months; **MN**, motor neuron; **MUNIX**, motor unit number index; **MSC-NTF**, mesenchymal stem cell-neurotrophic factor; **NF-κB**, nuclear factor kappa B; **NfL**, neurofilament light chain; **NPC**, neural progenitor cell; **NRF2**, nuclear factor erythroid 2-related factor 2; **PBR28-PET**, positron emission tomography using PBR28 TSPO ligand; **PDE**, phosphodiesterase; **PET**, positron emission tomography; **PI3K-Akt**, phosphatidylinositol 3-kinase-protein kinase B pathway; **PK**, pharmacokinetics; **pTDP-43**, phosphorylated TAR DNA-binding protein 43; **pts**, patients; **QALY**, quality-adjusted life year; **RCT**, randomized controlled trial; **Riluzole**, glutamate release inhibitor; **ROCK**, rho-associated coiled-coil containing protein kinase; **ROS**, reactive oxygen species; **sALS**, sporadic amyotrophic lateral sclerosis; **SAE**, serious adverse event; **SH-SY5Y**, human neuroblastoma cell line; **SLCP**, solid lipid curcumin particle; **SOD1**, superoxide dismutase 1; **SOD1^G93A^**, superoxide dismutase 1 Gly93Ala mutant mouse; **SUVR**, standardized uptake value ratio; **SVC**, slow vital capacity; **TBK1**, TANK-binding kinase 1; **TDP-43**, TAR DNA-binding protein 43; **TDP-43^A315T^**, TAR DNA-binding protein 43 Ala315Thr mutant mouse; **TMS**, transcranial magnetic stimulation; **TTNCS**, threshold tracking nerve conduction studies; **TUDCA**, tauroursodeoxycholic acid; **VEGF**, vascular endothelial growth factor; **WVE-004**, wave life sciences antisense oligonucleotide.

## Data Availability

No new data were created or analyzed in this study.

## Abbreviations

The following abbreviations are used in this manuscript:

15-LO	15-lipoxygenase
AAV	Adeno-associated virus
Aβ	Amyloid-beta
AChE	Acetylcholinesterase
AChE-I	Acetylcholinesterase inhibitor
AD	Alzheimer’s disease
ADAS	Alzheimer’s disease assessment scale
ADAM10	A disintegrin and metalloproteinase 10
ADCOMS	Alzheimer’s disease composite score
ADCS-ADL	Alzheimer’s disease cooperative study–activities of daily living
ADCS-MCI-ADL	Alzheimer’s disease cooperative study–activities of daily living scale for mild cognitive impairment
ADMET	Absorption, distribution, metabolism, excretion, and toxicity
ADSCs	Adipose-derived stem cells
AE	Adverse event
AIF	Apoptosis-inducing factor
ARIA	Amyloid-related imaging abnormalities
ALS	Amyotrophic lateral sclerosis
ALSFRS-R	Amyotrophic lateral sclerosis functional rating scale-revised
AMI	Active music intervention
AP2	Adaptor protein complex 2
APOE ε4	Apolipoprotein E
APP	Amyloid precursor protein
ATLIS	Accurate test of limb isometric strength
ASO	Antisense oligonucleotide
BAC	Bacterial artificial chromosome
BBB	Blood–brain barrier
BDNF	Brain-derived neurotrophic factor
BI	Barthel index
BMAA	β-N-methylamino-L-alanine
BM-MSCs	Bone marrow-derived mesenchymal stem cells
BPA	Bisphenol A
BuChE	Butyrylcholinesterase
BV-2	A murine microglial cell line
C9orf72	Chromosome 9 open reading frame 72
C9 iPSC-MNs	C9orf72 induced pluripotent stem cell-derived motor neurons
CBD	Corticobasal degeneration
CDR	Clinical dementia rating
CDR-SB	Clinical dementia rating-sum of boxes
cGAS	Cyclic GMP-AMP synthase
CHIEF-PD	Cholinesterase inhibitor to prevent falls in Parkinson’s disease
CIBIC-plus	Clinician’s interview-based impression of change plus caregiver input
C-SSRS	Columbia-suicide severity rating scale
Clau	(-)-Clausenamide
CNS	Central nervous system
CO	Carbon monoxide
COX	Cyclooxygenase
CSF	Cerebrospinal fluid
CYP19A1	Cytochrome P450 family 19 subfamily A member 1 (aromatase)
CYP3A4	Cytochrome P450 family 3 subfamily A member 4
DaT-SPECT	Dopamine transporter imaging with single-photon emission computed tomography
DEDRGs	Differential expression disulfidpotosis-related genes
DMNP	Dissolving microneedle patch
DRD2	Dopamine receptor D2
DRGs	Disulfidpotosis-related genes
EGFR	Epidermal growth factor receptor
EphA4	Ephrin type-A receptor 4
eQTLs	Expression quantitative trait loci
Fer-1	Ferrostatin-1
FGF21	Fibroblast growth factor 21
FN	Fibronectin
FT9	Fine-tuned 9-stage model
FTD	Frontotemporal dementia
FSTA	Fast skeletal muscle troponin activator
FTIR	Fourier transform infrared spectroscopy
FVC	Forced vital capacity
GAP43	Growth-associated protein 43
GAH	Galantamine hydrobromide
GB	Ginkgo biloba extract
GCase	Glucocerebrosidase
GDNF	Glial cell line-derived neurotrophic factor
GDS	Geriatric depression scale
GEO	Gene expression omnibus
GI	Gastrointestinal
GO	Gene ontology
GNPs	Graphene-based nanoparticles
GSH	Glutathione
GSK-3β	Glycogen synthase kinase 3 beta
GWAS	Genome-wide association studies
HEK293T	Human embryonic kidney 293T cells
HGF	Hepatocyte growth factor
HREI	High-resolution electron ionization
HPLC	High-performance liquid chromatography
HSP90AB1	Heat shock protein 90 alpha family class B member 1
HRR	Heart rate reserve
HTS	High-throughput screening
Hup A	Huperzine A
IC50	Half maximal inhibitory concentration
IGF-1	Insulin-like growth factor 1
IL	Interleukin
ILB^®®^	Low molecular weight dextran sulfate
iPSC	Induced pluripotent stem cell
IPDGC	International PD genomics consortium
IT	Intrathecal
ITC	Isothermal titration calorimetry
IV	Intravenous
KEAP1	Kelch-like ECH-associated protein 1
KEGG	Kyoto Encyclopedia of Genes and Genomes
LC	Liquid chromatography
LD/CD	Levodopa/carbidopa
LMDR	Wechsler memory scale logical memory delayed recall
LMW	Low molecular weight
LRRK2	Leucine-rich repeat kinase 2
MAO-B	Monoamine oxidase B
MAPK	Mitogen-activated protein kinase
MCI	Mild cognitive impairment
MCP-1	Monocyte chemoattractant protein-1
MDS-UPDRS	Movement disorder society-sponsored revision of the unified Parkinson’s disease rating scale
MEF	Mouse embryonic fibroblast
MEG	Magnetoencephalography
MIF	Macrophage migration inhibitory factor
MMSE	Mini-mental state examination
MOF	Metal–organic framework
MOVES-PD	Multicenter pharmacokinetics and interventional study in Parkinson’s Disease
MN	Motor neuron
MPTP	1-Methyl-4-phenyl-1,2,3,6-tetrahydropyridine
MR	Mendelian randomization
MRI	Magnetic resonance imaging
MS	Mass spectrometry
MSA	Multiple system atrophy
MSC-NTF	Mesenchymal stem cell-neurotrophic factor
MSN	Mesoporous silica nanoparticle
MUNIX	Motor unit number index
MTOR	Mechanistic target of rapamycin
MWM	Morris water maze
NeuMOF	Neutrophil-coated metal–organic framework
NFAT	Nuclear factor of activated T cells
NF-κB	Nuclear factor kappa B
NfL	Neurofilament light chain
NHIS	National health insurance service
NMDAR	N-methyl-D-aspartate receptor
NMR	Nuclear magnetic resonance
NO	Nitric oxide
NPC	Neural progenitor cell
NPI	Neuropsychiatric inventory
NPs	Nanoparticles
NRF2	Nuclear factor erythroid 2-related factor 2
PAAN	Parthanatos-associated apoptosis-inducing factor nuclease
PAANIB-1	Parthanatos-associated nuclease inhibitor-1
PACC	Preclinical Alzheimer cognitive composite
PARP	α poly (ADP-ribose) polymerase
PASADENA	Phase II trial of anti-α-synuclein antibody in early Parkinson’s disease
PBR28-PET	Positron emission tomography using PBR28 TSPO ligand
PCA	Protocatechuic acid
PC	Precuneus area
PD	Parkinson’s disease
PDD	Parkinson’s disease dementia
PDB	Protein data bank
PDE	Phosphodiesterase
PEP	Peptide inhibitor
PET	Positron emission tomography
PI3K-Akt	Phosphatidylinositol 3-kinase-protein kinase B pathway
PKCα	Protein kinase C α
PPMI	Parkinson’s progression markers initiative
pQTLs	Protein quantitative trait loci
PSD95	Postsynaptic density protein 95
PSEN	Presenilin
PSP	Progressive supranuclear palsy
pTDP-43	Phosphorylated TAR DNA-binding protein 43
qPCR	Real-time quantitative PCR
RAB24	Ras-related protein in brain 24
RCTs	Randomized controlled trials
RMI	Receptive music intervention
ROC	Receiver operating characteristic
ROCK	Rho-associated coiled-coil containing protein kinase
ROS	Reactive oxygen species
rTMS	Repetitive transcranial magnetic stimulation
SAE	Serious adverse events
sALS	Sporadic amyotrophic lateral sclerosis
SC	Subcutaneous
SEM	Scanning electron microscopy
SGF	Simulated gastric fluid
SH-SY5Y	Human neuroblastoma cell line
SIB	Severe impairment battery
SIF	Simulated intestinal fluid
SLCP	Solid lipid curcumin particle
SMDs	Standardized mean differences
SNAP	Synaptosomal-associated protein
SNCA	Synuclein alpha
SNP	Single nucleotide polymorphism
SOD	Superoxide dismutase
SSP	Semen Strychni Pulveratum
SUVR	Standardized uptake value ratio
SVC	Slow vital capacity
SYN1	Synapsin 1
SYT1	Synaptotagmin 1
TBK1	TANK-binding kinase 1
TDP-43	TAR DNA-binding protein 43
TEAEs	Treatment-emergent adverse events
TEM	Transmission electron microscopy
TH	Tyrosine hydroxylase
TMS	Transcranial magnetic stimulation
TNF	Tumor necrosis factor
TREM2	Triggering receptor expressed on myeloid cells-2
TS	Tinetti scale
TTNCS	Threshold tracking nerve conduction studies
TUDCA	Tauroursodeoxycholic acid
UA	Ursolic acid
VEGF	Vascular endothelial growth factor
WM	White matter
WT	Wild-type
WVE-004	Wave life sciences antisense oligonucleotide
XRD	X-ray diffraction
ZnO	Zinc oxide
